# Chemical Constituents from *Croton* Species and Their Biological Activities

**DOI:** 10.3390/molecules23092333

**Published:** 2018-09-12

**Authors:** Wen-Hui Xu, Wei-Yi Liu, Qian Liang

**Affiliations:** Key Laboratory for Forest Resources Conservation and Utilization in the Southwest Mountains of China, Ministry of Education, Southwest Forestry University, Kunming 650224, China; wenhuix001@hotmail.com (W.-H.X.); liuwy651vip@163.com (W.-Y.L.)

**Keywords:** *Croton* species, phytochemistry, biological activities, diterpenoids, cytotoxicity

## Abstract

The genus *Croton* belongs to the Euphorbiaceae family, which comprises approximately 1300 species. Many *Croton* species have been used as folk medicines. This review focuses on the chemical constituents from *Croton* species and their relevant biological activities, covering the period from 2006 to 2018. A total of 399 new compounds, including 339 diterpenoids, were reported. Diterpenoids are characteristic components of the *Croton* species. These isolated compounds exhibited a broad spectrum of bioactivities, including cytotoxic, anti-inflammatory, antifungal, acetylcholinesterase inhibitory, and neurite outgrowth-promoting properties. The present review provides a significant clue for further research of the chemical constituents from the *Croton* species as potential medicines.

## 1. Introduction

The genus *Croton* belongs to the Euphorbiaceae family, and contains approximately 1300 species of trees, shrubs, and herbs, which are widely distributed throughout tropical and subtropical regions of the world. Many *Croton* species have been used as folk medicines in Africa, south Asia, and south America, for the treatment of many diseases such as stomachache, abscesses, inflammation, and malaria [1,2,3]. The seeds of *C. tiglium*, which are well-known as “badou”, had been utilized as a traditional Chinese medicine to treat gastrointestinal disorders, intestinal inflammation, and rheumatism. The roots of *C. crassifolius*, known as “jiguxiang” in China, are mainly used as a traditional medicine for the treatment of stomachache and sore throat [3]. The genus *Croton* is abundant in diverse diterpenoids, including clerodane, tigliane, kaurane, labdane, cembrane, and pimarane, with a wide range of biological activities, such as cytotoxic, anti-inflammatory, and anti-microbial [1,2,3,4,5]. Due to their great structural diversity and broad relevant bioactivities, *Croton* species have attracted increasing research attention. Several authors have provided reviews about the chemical constituents and biological activities of Croton species. A review came out in 2006 regarding clerodane diterpenes isolated from *Croton* species, their ^13^C-NMR spectroscopic data, and biological activities [2]. In 2007, a comprehensive review on the traditional uses, chemistry, and pharmacology of *Croton* species was published [1]. In 2013, anticancer and antioxidant activities of extracts and pure compounds from several *Croton* species were reviewed [4]. Five review articles were published in recent years which focused on ethnopharmacological uses, phytochemistry, and pharmacology of a single *Croton* species [6,7,8,9,10]. In the last decade, there has been a dramatic progress in the chemical constituents and relevant biological activities of *Croton* species. However, so far, no comprehensive review has been published since 2007. In the present review, we summarize systematically the research advances on the new chemical constituents and their biological activities of *Croton* species reported in the literature, as found on Web of Science, Google Scholar, PubMed, and SciFinder, from 2006 to March 2018, with the aim of providing a basis for further research of natural product drug discovery.

## 2. Chemical Constituents

**Figure 1:**
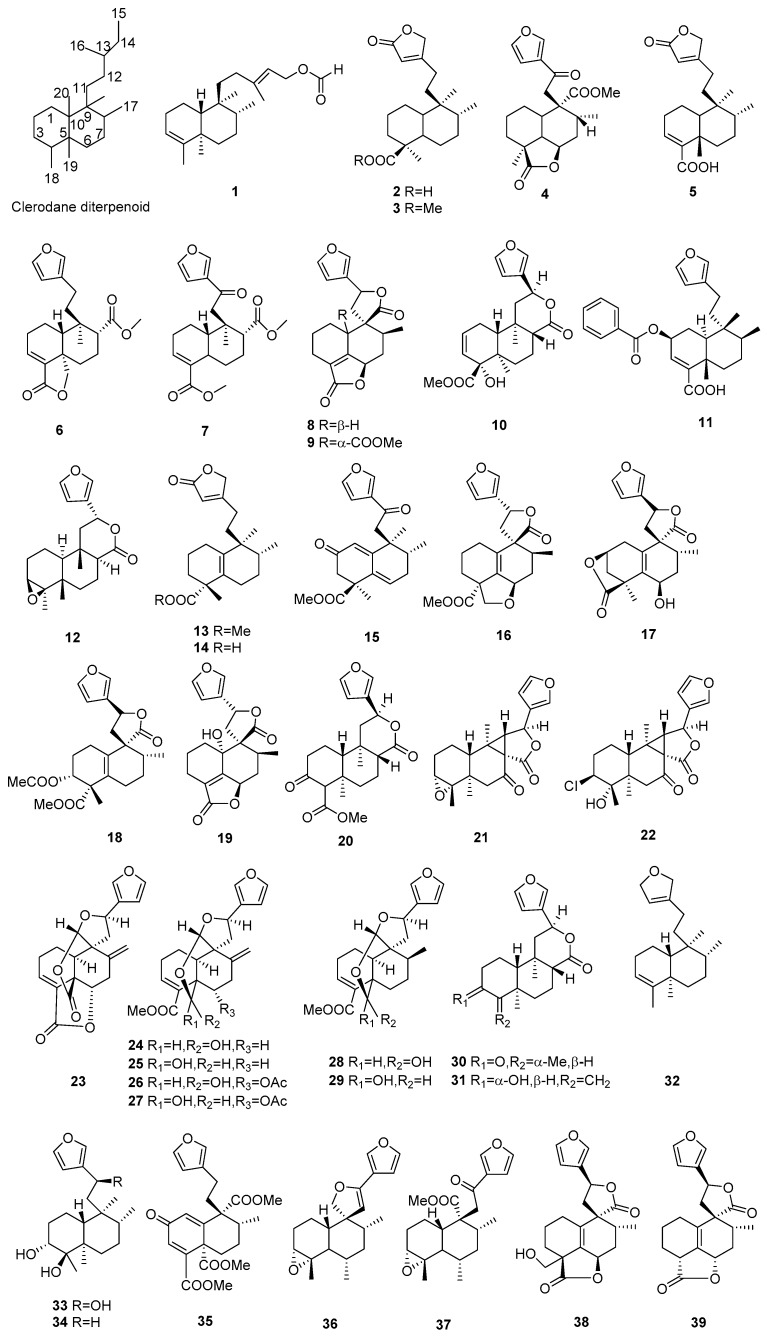

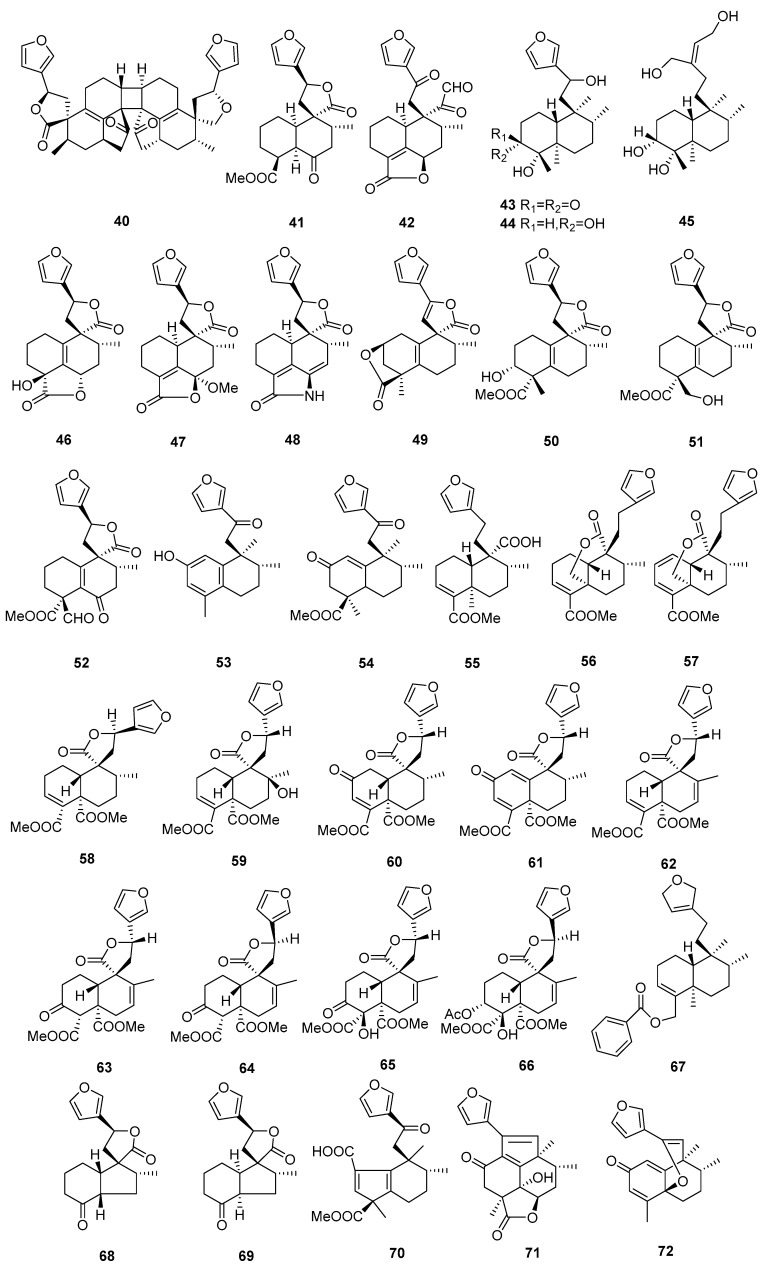

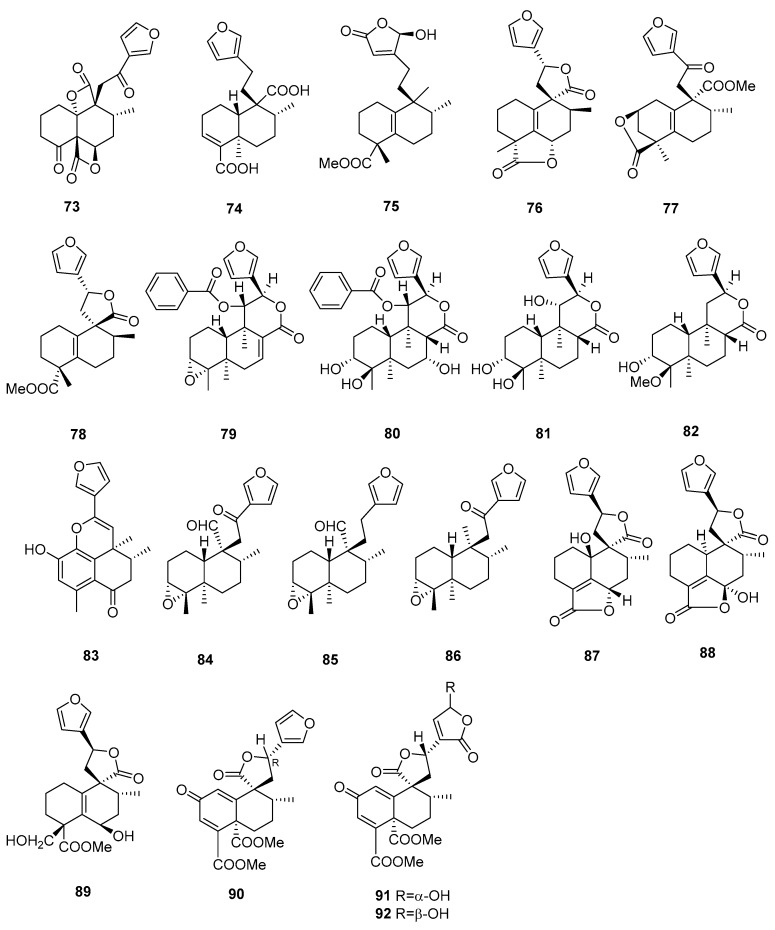
Clerodane type diterpenoids from the genus *Croton*.

**Figure 2:**
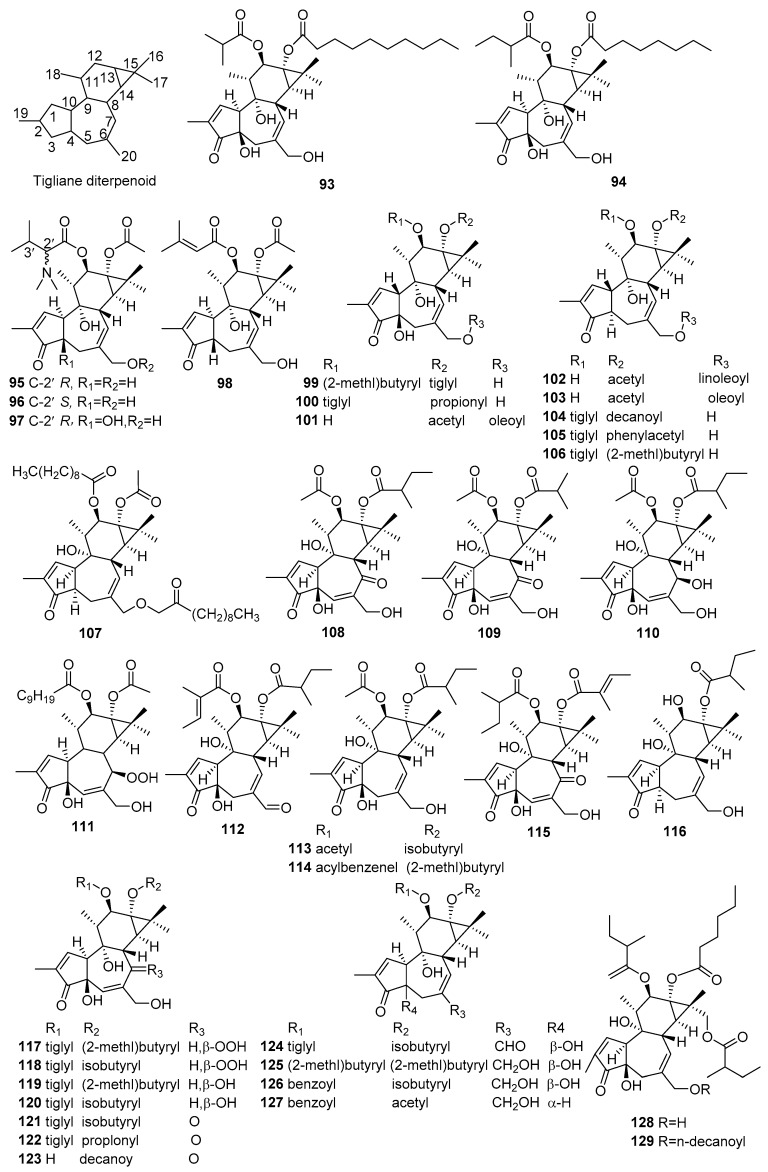

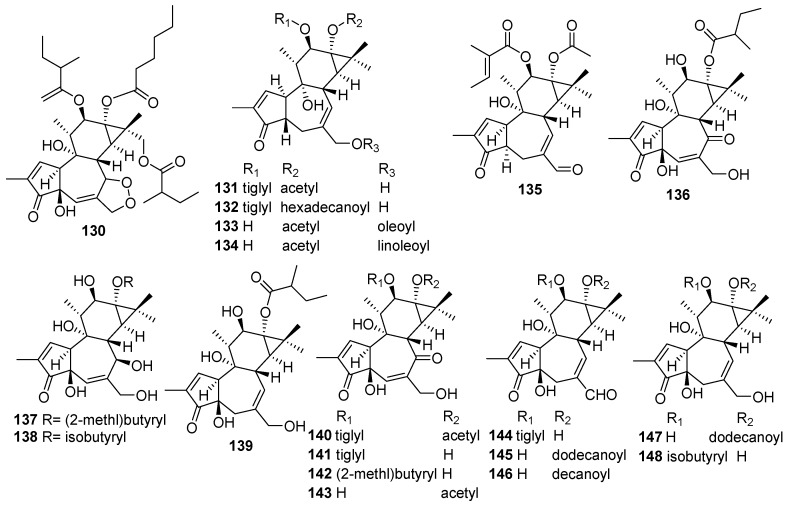
Tigliane type diterpenoids from the genus *Croton*.

**Figure 3:**
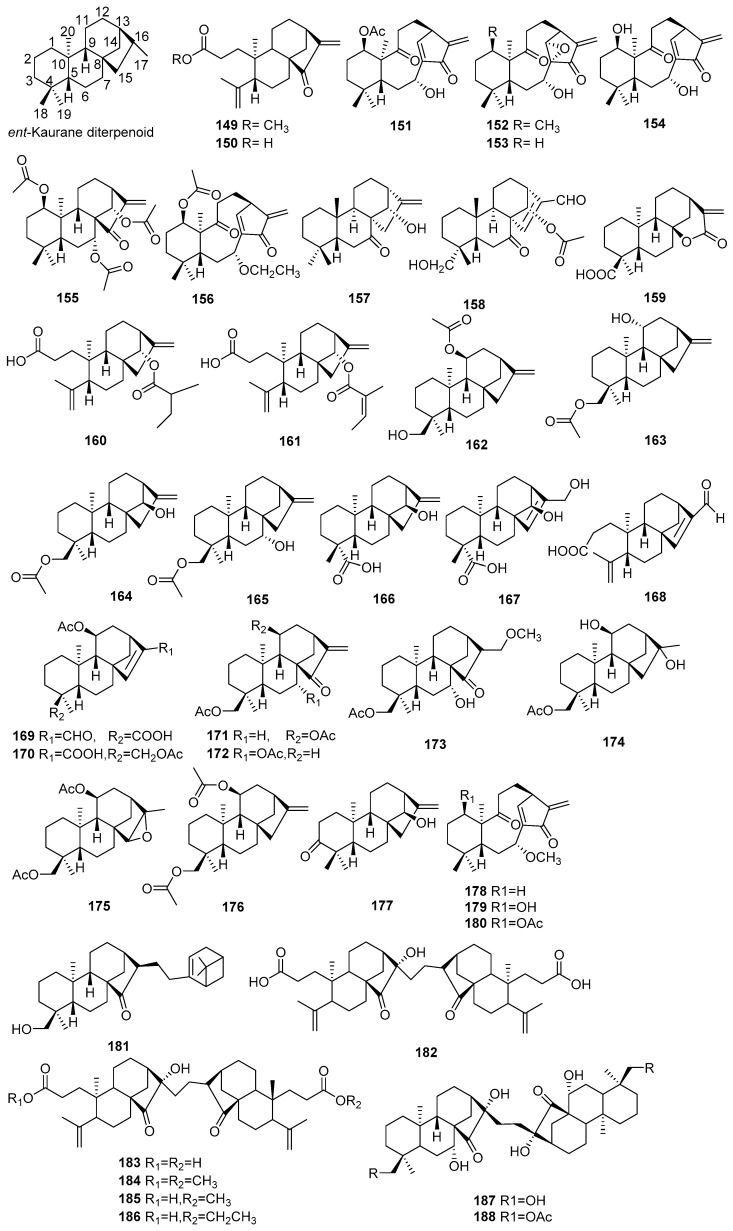
Kaurane type diterpenoids from the genus *Croton* 1.

**Figure 4:**
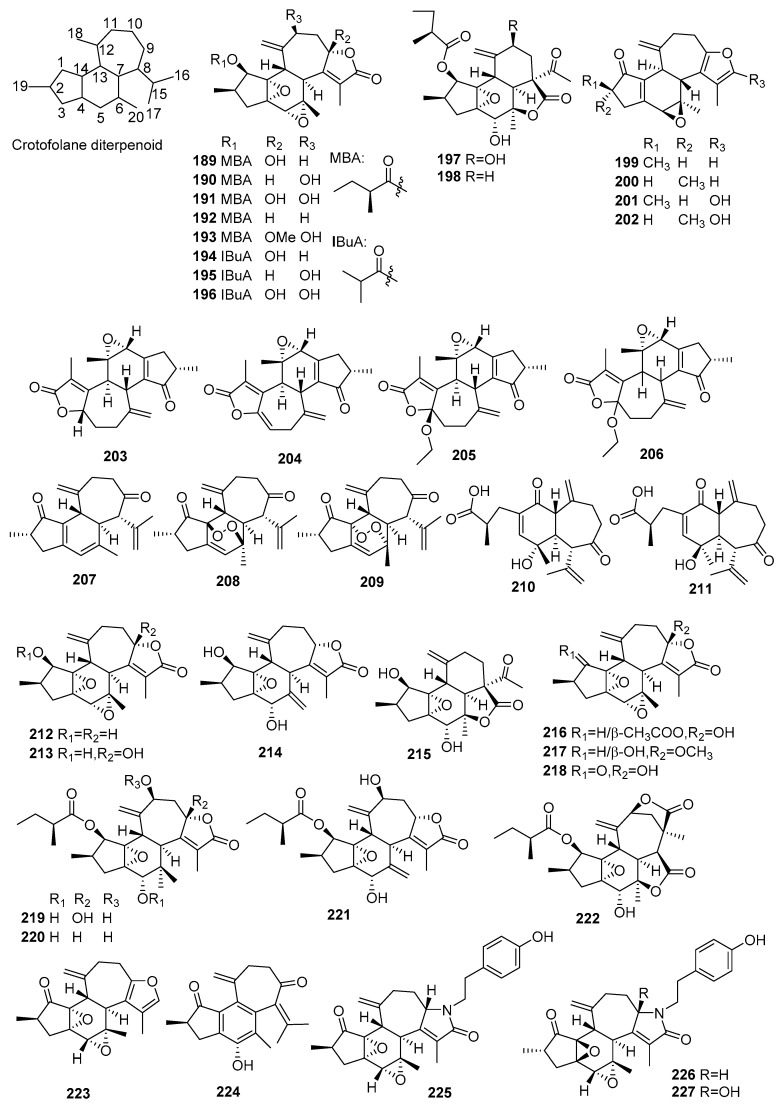
Crotofolane type diterpenoids from the genus *Croton*.

**Figure 5:**
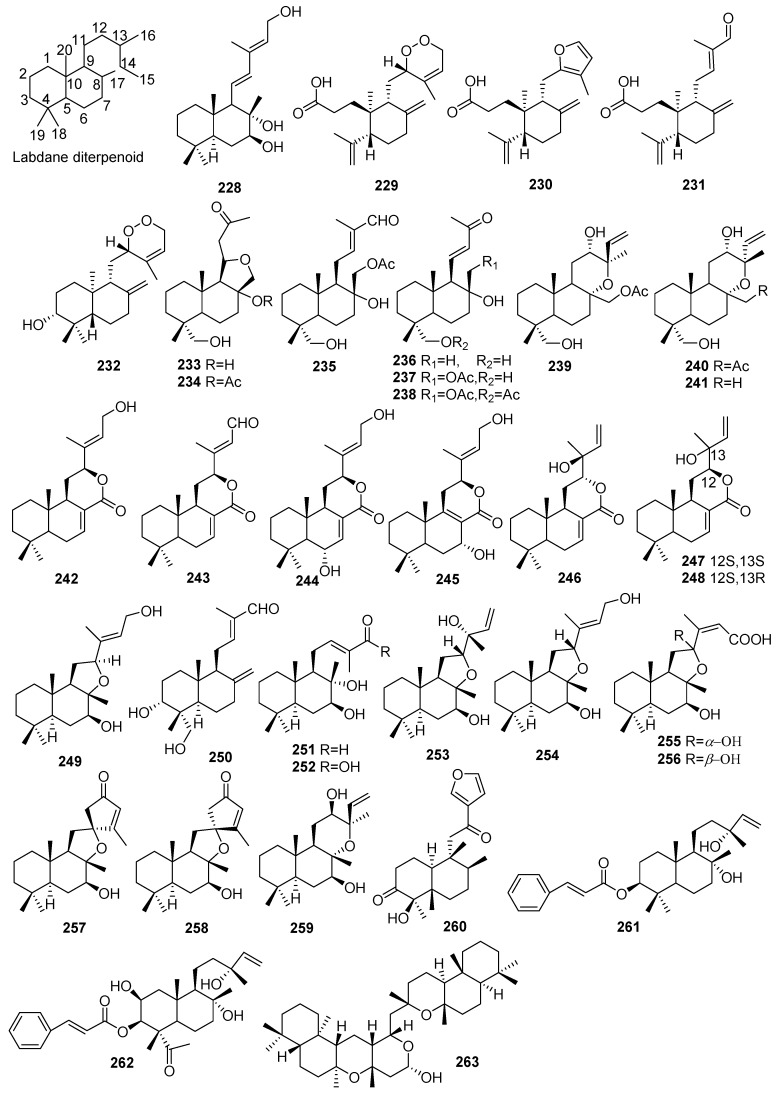
Labdane type diterpenoids from the genus *Croton*.

**Figure 6:**
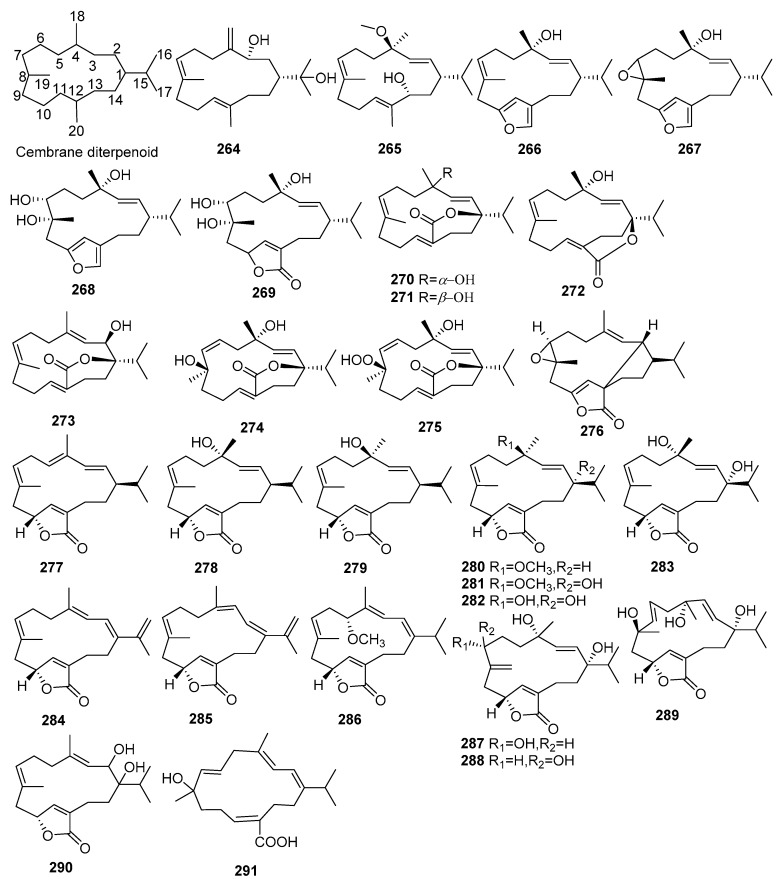
Cembrane type diterpenoids from the genus *Croton*.

**Figure 7:**
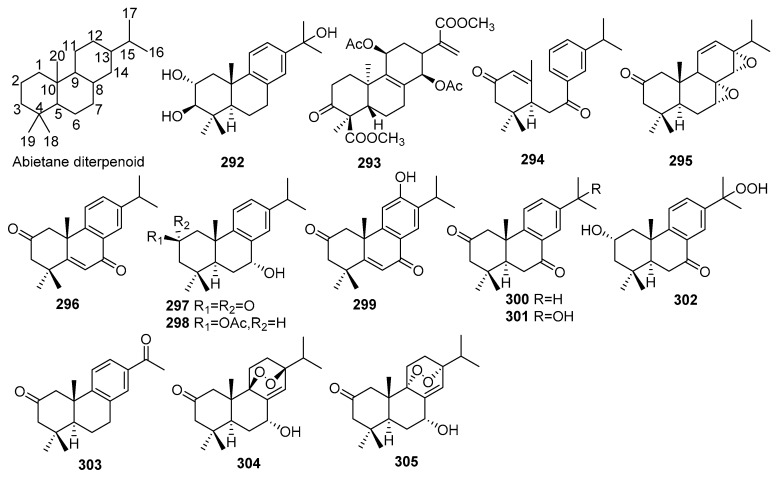
Abietane type diterpenoids from the genus *Croton*.

**Figure 8:**
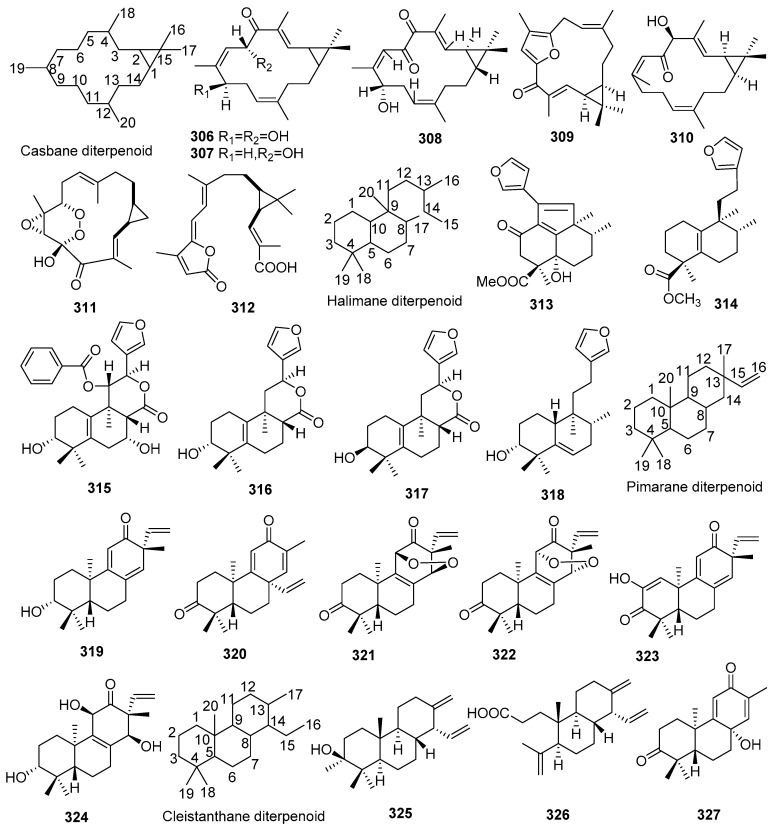
Casbane, Halimane, Pimarane and Cleistanthane type diterpenoids from the genus *Croton*.

**Figure 9:**
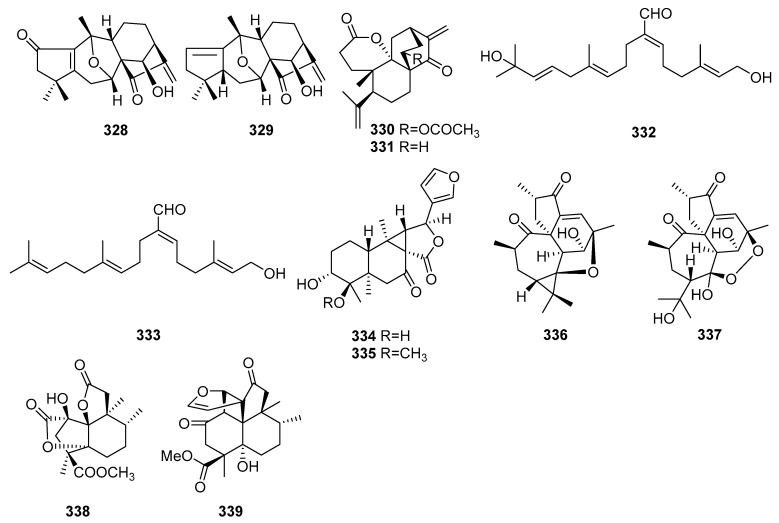
Grayanane, Atisane, Phytane, Laevinane type diterpenoids and Meroditerpenoids from the genus *Croton*.

**Figure 10:**
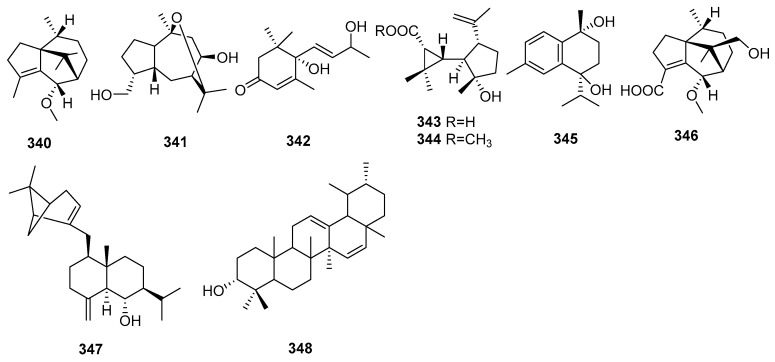
Sesquiterpenoids, Sesterterpenoid and Triterpenoid from the genus *Croton*.

**Figure 11:**
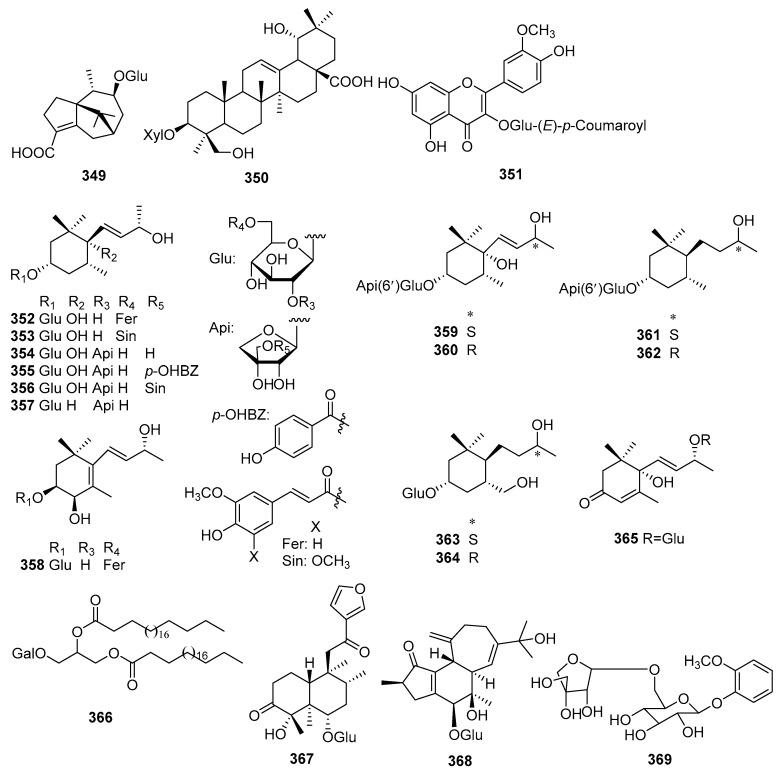
Glycosides from the genus *Croton*.

**Figure 12:**
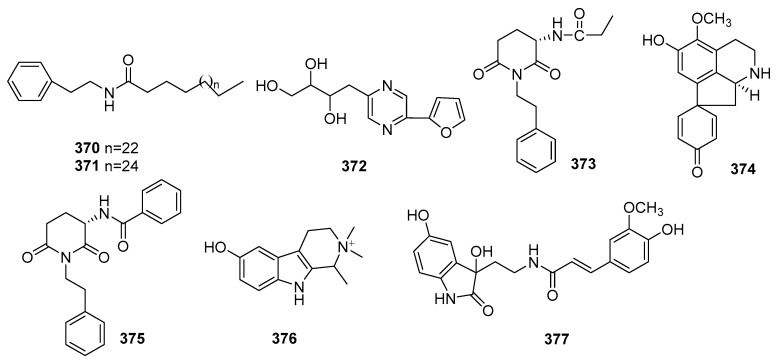
Alkaloids from the genus *Croton*.

**Figure 13:**
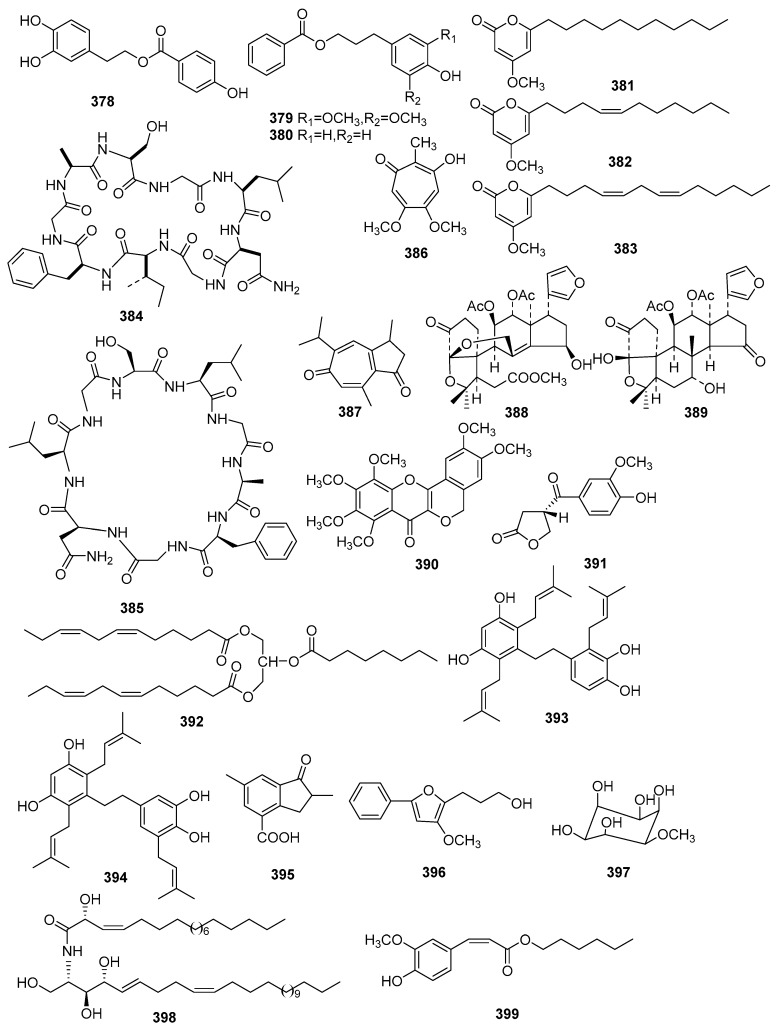
Miscellaneous compounds from the genus *Croton*.

To date, 399 new compounds have been isolated and identified from *Croton* species, including 339 diterpenoids (**1**–**339**), seven sesquiterpenoids (**340**–**346**), one sesterterpenoid (**347**), one triterpenoid (**348**), 21 glycosides (**349**–**369**), eight alkaloids (**370**–**377**), three benzoate derivatives (**378**–**380**), three pyran-2-one derivatives (**381**–**383**), two cyclopeptide (**384**, **385**), two tropone derivatives (**386**, **387**), two limonoids (**388**, **389**), and ten miscellaneous compounds (**390**–**399**). Their structures, molecular formula, names, corresponding sources, and references are summarized in Figure 1, Figure 2, Figure 3, Figure 4, Figure 5, Figure 6, Figure 7, Figure 8, Figure 9, Figure 10, Figure 11, Figure 12 and Figure 13 and Table 1, Table 2, Table 3, Table 4, Table 5, Table 6, Table 7, Table 8, Table 9, Table 10, Table 11, Table 12, Table 13, Table 14, Table 15, Table 16, Table 17, Table 18, Table 19, Table 20, Table 21, Table 22, Table 23, Table 24, Table 25, Table 26 and Table 27.

### 2.1. Diterpenoids

Phytochemical investigations on *Croton* species revealed the predominant secondary metabolites as diterpenoids, including clerodane, tigliane, kaurane, crotofolane, labdane, cembrane, abietane, casbane, halimane, pimarane, cleistanthane, grayanane, atisane, phytane, and laevinane diterpenoids. Three hundred & thirty-nine new diterpenoids (**1**–**339**) were reported from *Croton* species.

#### 2.1.1. Clerodanes

Ninety-two new clerodane diterpenoids (**1**–**92**) were isolated from *Croton* species, including two clerodane diterpenoid with acyclic at C-9s, eight clerodane diterpenoids with butenolide at C-9, and 82 furan-clerodane diterpenoids [11]. Their structures, molecular formula, names, corresponding sources, and references are listed in Figure 1 and Table 1. Two new clerodane diterpenoids with acyclic side chain at C-9, *ent*-3,13*E*-clerodadiene-15-formate (**1**) and 3*α*,4*α*,15,16-tetrahydroxy-*ent*-*neo*-cleroda-13*E*-ene (**45**), were isolated from the roots of *C*. *sylvaticus* [12] and the roots of *C. limae* [13], respectively. Eight new clerodane diterpenoids with butenolide at C-9 (**2**, **3**, **5**, **13**, **14**, **75**, **91**, **92**) were obtained from three *Croton* species (*C. crassifolius*, *C. glabellus*, and *C. oligandrus*) [14,15,16,17,18]. Furan-clerodane diterpenoids are abundant in *Croton* species, and 82 new ones were isolated from different Croton species. For example, Centrafricine I (**4**) from *C*. *mayumbensis* was a new furan-clerodane diterpenoid with a 6, 18-γ-lactone ring [19]. Two novel rearranged *ent*-clerodane diterpenoids Laevinoids A, B (**21**, **22**) containing an unusual 3/5 bicyclic ring were obtained from the branches and leaves of *C*. *laevigatus*; **22** represents the first chlorinated example of the clerodane family [20]. Compounds (**23**–**27**) bearing a C-19/C-20 six-membered ring were identified from *C. laui* [21]. Phytochemical investigations on three *Croton* species (*C. oblongifolius*, *C. yanhuii*, and *C. hypoleucus*) afforded six new furan-clerodanoids (**12**, **36**, **37**, **84**–**86**) with a 3,4-epoxy moiety [22,23,24]. Crotoeurins A–C (**40**–**42**) were found from the twigs and leaves of *C. euryphyllus*. Among them, crotoeurin A (**40**) was a *nor*-clerodane diterpenoid dimer with a unique cyclobutane ring via a [2 + 2] cycloaddition [25]. Three new furan-clerodane diterpenoids, cracroson A–C (**46**–**48**) were obtained from *C. crassifolius*, while cracroson C (**48**) represents the first example of a clerodane diterpenoid alkaloid [26]. Twelve new *ent*-clerodanoids (**55**, **66**) were isolated from the roots of *C. megalocarpoides*. Among them, compounds (**58**–**66**) possessed 9, 12-γ-lactone ring [27]. Investigation on the roots of *C. crassifolius* afforded eight new clerodanoids, crassins A−H (**68**–**75**). Among them, crassins A–B (**68**, **69**) represents ring B rearranged clerodanoids, whereas crassins C (**70**) was ring A rearranged one [17]. One new *nor*-clerodane diterpenoid, norcrassifolin (**83**), with a 1,12-lactone six-membered ring, was isolated from *C. crassifolius* [28].

#### 2.1.2. Tiglianes

Fifty-six new tigliane diterpenoids (**93**–**148**) were reported from *Croton* species. Their structures, molecular formula, names, corresponding sources, and references are collected in Figure 2 and Table 2. Investigations on the aerial parts of *C. ciliatoglandulifer* produced four new tiglianoids (**95**–**98**). Among them, tiglianoids (**95**–**97**) possess a *N*,*N*-dimethyl moiety at 2′position [41]. Alienusolin (**107**) and compound (**111**) were obtained from the roots and the leaves of *C. alienus* and the leaves of *C. mauritianus,* respectively [42,43]. The twigs and leaves of *C. caudatus* produced three new tiglianoids, crotusins A–C (**128**–**130**) [44]. Tigliane diterpenoids were abundant in *C. tiglium*, other 47 new ones (**93**, **94**, **99**–**106**, **108**–**110**, **112**–**127**, **131**–**148**) were isolated from *C. tiglium* [45,46,47,48,49,50,51,52]. Among them, compound (**112**) was the first tiglianoid with the C20-aldehyde group [48].

#### 2.1.3. Kauranes

Fourty new kaurane diterpenoids (**149**–**188**) were found from *Croton* species. Their structures, molecular formula, names, corresponding sources, and references are listed in Figure 3 and Table 3. Five new 3,4-*seco ent*-kauranes (**149**–**150**, **160**–**161**, **168**) were isolated from *C. caracasana* [53], *C. megistocarpus* [54], and *C. oblongifolius* [55], respectively. Investigations on *C. kongensis* afforded eight new 8,9-*seco-ent*-kaurane diterpenes (**151**–1**54**, **156**, **178**–**180**) [56,57,58,59]. Compound **181**, one new kaurane bearing a monoterpene unit at C-16, was found from *C. limae* [35]. From the stems of *C. micans*, five new 3,4-*seco*-*ent*-kaurene dimers (**182**–**186**) were isolated [60], while other two dimeric *ent*-kaurane diterpenoids (**187**–**188**) were elucidated from *C*. *tonkinensis* [61].

#### 2.1.4. Crotofolanes

Thirty-nine new crotofolane diterpenoids (**189**–**227**) were obtained from *Croton* species. Their structures, molecular formula, names, corresponding sources, and references are summarized in Figure 4 and Table 4. Twenty-four new crotofolane diterpenoids (**189**–**198**, **212**–**222**, **225**–**227**) were isolated from *C*. *caracasanus* [68,69,70,71]. Among them, three new crotofolane diterpenoid alkaloids, cascarinoids A–C (**225**–**227**), were firstly found. Investigations on *C. argyrophyllus* gave four new crotofolanes (**199**–**202**) [72]. Crotocarasin A–D (**203**–**206**) were isolated from the stems of *C. caracasanus* [73]. Five new 1, 14-*seco*-crotofolanes from *C. insularis* were obtained [74], while *C. dichogamus* yielded crotodichogamoin A–B (**223**–**224**) [75].

#### 2.1.5. Labdanes

Thirty-six new labdane diterpenoids (**228**–**263**) were isolated from *Croton* species. Their structures, molecular formula, names, corresponding sources, and references are collected in Figure 5 and Table 5 12 new labdanes (**228**, **249**–**259**) were isolated from *C*. *laui* [21,76,77]. From the leaves of *C*. *stipuliformis*, three 3,4-*seco*-*ent*-labdanes (**229**–**231**) and one *ent*-labdane (**232**) were obtained [78]. Investigation of *C*. *laevigatus* led to the isolation of 16 new labdanes (**233**–**248**). Among them, crotonlaevins A–B (**233**, **234**), represents rare labdanes with a dodecahydronaphtho [1,2-c] furan moiety [79]. Three new labdane diterpenoids (**260**–**262**) were found from *C. jacobinensis* [77] and *C. decalvatus* [80], respectively. Bicrotonol A (**263**), one dimeric labdane-type diterpenoid, was obtained from the roots of *C*. *crassifolius* [81].

#### 2.1.6. Cembranes

A total of 28 new cembrane diterpenoids (**264**–**291**) were obtained from *Croton* species. Their structures, molecular formula, names, corresponding sources, and references are listed in Figure 6 and Table 6. launine O-P (**264**, **265**), two new cembranes, were reported from the aerial parts of *C*. *laui* [76]. Investigations on the stem bark of *C. oblongifolius* afforded four new furanocembranoids (**266**–**269**) [83]. laevigatlactones A–F (**270**–**275**), six new cembranoids possessing a rare six-membered lactone moiety attached to C-1 and C-20, were firstly isolated from *C*. *laevigatus* [84]. 14 new cembranoids (**276**–**289**) were found from *C*. *gratissimus* [85,86]. Among them, compound **276** was first example of a 2,12-cyclocembranolide. The leaves of *C*. *longissimus* produced two new cembranes (**290**, **291**) [87].

#### 2.1.7. Abietanes

Fourteen new abietane diterpenoids (**292**–**305**) were isolated from *Croton* species. Their structures, molecular formula, names, corresponding sources, and references are collected in Figure 7 and Table 7. Two new abietanes (**292**, **293**) were obtained from *C. megalocarpoides* [27], and *C. argyrophylloides* [63], respectively. Investigation of *C*. *caudatus* led to the isolation of 5 new abietanes (**294**–**298**). Among them, crotontomentosin A (**294**) was a 9,10-*seco* abietane [88]. Crotolaevigatones A–G (**299**–**305**), 7 new abietanes were found from the twigs and leaves of *C. laevigatus*, and compounds (**304**, **305**) possessed a 9,13-epidioxy moiety [89].

#### 2.1.8. Casbanes

Seven new casbane diterpenoids (**306**–**312**) were found from *Croton* species. Their structures, molecular formula, names, corresponding sources, and references are summarized in Figure 8 and Table 8. Five new casbane s (**306**–**310**) were reported from *C. nepetaefolius* [90], and *C. argyrophyllus* [72,91], respectively. Investigations on the stem bark of *C. insularis* afforded two new casbanes, EBC-324 (**311**) and EBC-329 (**312**). Among them, EBC-329 (**312**) represented the first natural *seco*-casbane diterpene, while EBC-324 (**311**) was the first endoperoxide casbane [92].

#### 2.1.9. Halimanes

Six new halimane diterpenoids (**313**–**318**) were reported from *Croton* species. Their structures, molecular formula, names, corresponding sources, and references are collected in Figure 8 and Table 9. Investigations on the stem bark of *C. oblongifolius* afforded two new cleistanthanes (**325**, **326**). Among them, compound **326** was a 3,4-*seco* cleistanthane [93]. One new bis-*nor*-cleistanthane diterpenoid (**327**), was found from the twigs and leaves of *C. caudatus* [94].

#### 2.1.10. Pimaranes

Six new pimarane diterpenoids (**319**–**324**) were obtained from *Croton* species. Their structures, molecular formula, names, corresponding sources, and references are listed in Figure 8 and Table 10. All six new pimaranes (**319**–**324**) were isolated from *C. insularis* [96,97]. Among them, compound **319** was an important biosynthetic intermediate.

#### 2.1.11. Cleistanthanes

Three new cleistanthane diterpenoids (**325**–**327**) were ioslated from *Croton* species. Their structures, molecular formula, names, corresponding sources, and references are collected in Figure 8 and Table 11. Investigations on the stem bark of *C. oblongifolius* afforded two new cleistanthanes (**325**, **326**). Among them, compound **326** was a 3,4-*seco* cleistanthane [93]. One new bis-*nor*-cleistanthane diterpenoid (**327**), was found from the twigs and leaves of *C. caudatus* [94].

#### 2.1.12. Grayananes, Atisanes, Phytanes, Laevinanes and Meroditerpenoids

From the leaves of *C. tonkinensis*, two new rare grayanane diterpenoids, crotonkinensins A (**328**) and B (**329**), were isolated [100]. Two new 3,4-*seco* atisane diterpenoids, crotobarin (**330**) from *C. barorum* and crotogoudin (**331**) from *C. goudotii*, were found [101]. Investigations on the aerial parts of *C. laui* gave two new phytane diterpenoids (**332**, **333**) [37]. Two new laevinane diterpenoids, crolaevinoid G (**334**) and H (**335**), were obtained [39]. Two new meroditerpenoids, steenkrotin A (**336**) and B (**337**), containing new carbon skeletons, were isolated from the leaves of *C. steenkampianus* [102]. From the the roots of *C. crassifolius*, two new meroditerpenoids, norcrassin A (**338**) and cracroson D (**339**), were reported [35,69]. Among them, norcrassin A (**338**) possessing a new carbon skeleton with a 5/5/5/6 tetracyclic system, was a C16 tetranorditerpenoid, while *c*racroson D (339) featured a new skeleton with a rare cyclobutane ring. Their structures, molecular formula, names, corresponding sources, and references are listed in Figure 9 and Table 12, Table 13, Table 14, Table 15 and Table 16.

### 2.2. Sesquiterpenoids, Sesterterpenoids and Triterpenoids

Seven new sesquiterpenoids (**340**–**346**), one sesterterpenoid (**347**) and one triterpenoid (**348**) were ioslated from *Croton* species. Their structures, molecular formula, names, corresponding sources, and references are summarized in Figure 10 and Table 17, Table 18 and Table 19. From *C. muscicarpa*, one new patchoulane sesquiterpenoid (**340**) was obtained [103]. A guaiane sesquiterpenoid (**341**) was isolated from *C. regelianus* [94]. Investigations on the leaves of *C. pedicellatus* afforded a bis-*nor*-sesquiterpenoid (**342**) [104]. Two rare sesquiterpenoid, Crocrassins A (**343**) and B (**344**) having cyclopropylcyclopentane moiety, were reported [105]. Other two sesquiterpenoids, 1,3,5-cadinatriene-(7*R*,10*S*)-diol (**345**) and cracroson H (**346**) were found from *C. dichogamus* [75], and *C. crassifolius* [40], respectively. One rare sesterterpenoid, pseudopulchellol (**347**), was isolated from the leaves of *C. pseudopulchellus* [106]. From the root of *C. bonplandianum*, a new ursane triterpenoid (**348**) was obtained [107].

### 2.3. Glycosides

Twenty-one new glycosides (**349**–**369**) were ioslated from *Croton* species. Their structures, molecular formula, names, corresponding sources, and references are collected in Figure 11 and Table 20. From *C. crassifolius*, a patchoulane sesquiterpenoid glycoside (**349**), an isocrotofolane glucoside (**368**), and a phenolic glycoside (**369**) were reported [69,108]. Compound **350**, isolated from *C. lachnocarpus*, was the first triterpenoid glucoside reported from the genus *Croton* [109]. A new flavone glucoside (**351**) was found from the leaves of *C. zambesicus* [110]. Investigations on the leaves of *C. cascarilloides* and *C. oblongifolius* afforded 13 new megastigmane glycosides, crotonionosides A–G (**352**–**358**) and Oblongionosides A–F (**359**–**364**) [111,112]. One new bis-*nor*-sesquiterpenoid glycoside (**365**) was isolated from *C. pedicellatus* [104]. One new diglyceride galactoside (**366**) and one new clerodane glucoside (**367**) were obtained from *C. sparsiorus* [113], and *C. limae* [35], respectively.

### 2.4. Alkaloids

Eight new alkaloids (**370**–**377**) were reported from *Croton* species. Their structures, molecular formula, names, corresponding sources, and references are listed in Figure 12 and Table 21. From *C. sparsiflorus*, two new amide alkaloids crotamides A (**370**) and B (**371**), and one new proaporphine alkaloid, crotsparsidine (**374**) were isolated [96,114]. One new pyrazine derivative, crotonine (**372**) was obtained from the leaves of *C. tiglium* [97]. Investigations on *C. cascarilloides* afforded a new glutarimide alkaloid, crotonimide C (**375**) [42]. Other three new alkaloids (**373**, **376**–**377**) were found from C. pullei, C. heliotropiifolius, and C. echioides, respectively [115,116,117]. 

### 2.5. Benzoate Derivatives, Pyran-2-One Derivatives, Cyclicpeptides, Tropone Derivatives and Limonoids

Three benzoate derivatives (**378**–**380**) were isolated from *C. sylvaticus* and *C. hutchinsonianus* [118,119]. Investigations on *C. crassifolius* afforded three new pyran-2-one derivatives, crotonpyrone A (**381**), B (**382**) and C (**383**) [120,121]. Two cyclicpeptides (**384**, **385**) were obtained from *C. gossypifolius* and *C. urucurana* [122,123], while two tropone derivatives (**386**, **387**) were isolated from *C. zehntneri* and *C. argyroglossum* [124,125]. From the root bark of *C. jatrophoides*, two new limonoids, musidunin (**388**) and musiduol (**389**), were found [126]. Their structures, molecular formula, names, corresponding sources, and references are collected in Figure 13 and Table 22, Table 23, Table 24, Table 25 and Table 26. 

### 2.6. Miscellaneous Compounds

Flavonoids, lignans, and other types of 10 compounds were also isolated from *Croton* species. Their structures, molecular formula, names, corresponding sources, and references are collected in Figure 13 and Table 27. From the stems of *C. caudatus*, one new flavone, crotoncaudatin (**390**), was isolated [127]. A new *nor*-lignan (**391**) was obtained from the twigs and leaves of *C. kongensis* [67]. Investigations on *C. laevifolius* gave two new prenylated dihydrostilbenes, laevifolin A (**393**), B (**394**) and one new aromatic compound (**399**) [89,128]. A long chain linear ester, lobaceride (**392**) was isolated from the twigs and leaves of *C. lobatus* [129]. One indanone derivative (**395**) was found from the leaves of *C. steenkampianus* [102], while a trisubstituted furan derivative (**396**) was isolated from the bark of *C. oblongifolius* [22]. From *C. sparsiflorus*, an inositol, sparsifol (**397**), and a sphingolipid, sparsioamide (**398**), were obtained [96,113].

## 3. Biological Activities

Compounds isolated from *Croton* species exert a wide range of biological activities, including cytotoxic, anti-inflammatory, antifungal, acetylcholinesterase inhibitory, and neurite outgrowth-promoting activities.

### 3.1. Cytotoxic Activity

The anti-tumor activity of many plants from the *Croton* species have been reported. Therefore, the cytotoxicity of the isolated compounds is the most commonly studied bioactivity. The cytotoxic activities of the isolated compounds from the *Croton* species are listed in Table 28. Four new tigliane diterpene esters (**135**–**137**, **139**) from the leaves of *C*. *tiglium*, exhibited most potent cytotoxic activity against K562 cell line with IC_50_ values of 0.03, 0.03, 0.07 and 0.05 μM, respectively [51].

### 3.2. Anti-Inflammatory Activity

Bioassay-guided fractionation of the aerial parts of *C. ciliatoglandulifer* led to the isolation of tigliane diterpenoids **95**, **97**, which inhibited the enzymes cyclooxygenases-1 (IC_50_, 0.001, and 1.0 μM, respectively) and cyclooxygenases-2 (IC_50_, 2.2 μM, for compound **95**) [41]. A tigliane diterpenoid (**114**) was isolated from the branches and leaves of C*. tiglium*, which displayed moderate inhibition of the enzymes COX-1 and COX-2, with IC_50_ values of 0.14 and 8.5 μM, respectively [48]. crotonkinin A (**157**), isolated from *C. tonkinensis*, showed anti-inflammatory effect on LPS-induced iNOS-dependent NO production and NOX-dependent ROS production in microglial cells (IC_50_, 46.2 ± 3.1 μM in NOS; maximum inhibition of NOX activity at 50 μM, 11.2%) [62]. Eight *ent*-kauranes (**169**–**176**) from *C. tonkinensis* exhibited the anti-inflammatory potential for inhibition of superoxide Anion generation and elastase release. Among them, crotonkinins F (**172**) displayed significant inhibition of superoxide anion generation (IC_50_, 2.88 ± 0.52 μM) and elastase release (IC_50_, 4.44 ± 1.45 μM) [66]. Labdane diterpenoids **251**, **254** and **257**, **258**, isolated from the aerial parts of *C. laui*, were found to show anti-inflammatory activities in LPS-stimulated RAW 264.7 cells with IC_50_ values in the range 42.73–93.04 μM [82]. Two grayanane diterpenoids, crotonkinensins A (**328**) and B (**329**) from the leaves of *C. tonkinensis*, were reported to decrease the LPS-induced COX-2 promoter activity in Raw 264.7 cells with IC_50_ values of 7.14 ± 0.2 and 5.49 ± 0.2 μM, respectively [100]. Two benzoate derivatives (**379**, **380**) were obtained from *C. hutchinsonianus*. Compound **379** showed significant activity against COX-1 (IC_50_, 4.95 ± 0.58 μg/mL) and COX-2 (IC_50_, 2.11 ± 1.3 μg/mL), while compound **380** (IC_50_, 1.88 ± 0.17 μg/mL) preferentially inhibited COX-2 [119].

### 3.3. Antifungal Activity

Two benzoate derivatives (**379**–**380**) were isolated from *C. hutchinsonianus*, and exhibited antifungal activity against *Candida albicans* (IC_50_, 11.41 ± 1.44, and 5.36 ± 0.01 μg/mL, respectively) [119]. Ursane triterpenoid (**348**) from the root of *C. bonplandianum*, displayed the antifungal activity against *Calletotricheme camellie* (IC_50_, 10 μg/mL), *Fussarium equisitae* (IC_50_, <15 μg/mL), *Alterneria alternata* (IC_50_, 10 μg/mL), *Curvularia eragrostidies* (IC_50_, <10 μg/mL) and *Colletorichum gloeosporiodes* (IC_50_, 15 μg/mL) [107].

### 3.4. Acetylcholinesterase Inhibitory Activity

An indole alkaloid derivative **376**, isolated from the leaves of *C. heliotropiifolius*, exhibited the acetylcholinesterase inhibitory activity with IC_50_ values of 17.8 ± 0.6 μM [116]. Compund **378** from *C. sylvaticus*, also displayed the same activity [118].

### 3.5. Neurite Outgrowth-Promoting Activity

Two clerodane diterpenoids, crotonpenes A (**36**) and B (**37**) were isolated from *C. yanhuii*, which markedly increased the NGF (20 ng/mL)-induced proportion of neurite bearing cells by 59%, and 47% at 15 μM, respectively [23]. Crotoeurins A–C (40–42) obtained from *C. euryphyllus*, were found to display neurite outgrowth-promoting activity on NGF mediated PC12 cells at concentration of 10 μM. The percentages of neurite-bearing cells were 9.72%, 14.90%, and 7.14%, respectively [25].

### 3.6. Other Activities

Besides the above activities, other biological activities have also been reported. Crotonolide G (**32**), from the aerial parts of *C. laui*, was found to exhibit potent antibacterial activity (MIC, 43.4 μM) against four strains of Gram-positive bacteria, namely, *Staphylococcus aureus*, *Staphylococcus epidermidis*, *Micrococcus luteus*, and *Bacillus subtilis* [21]. Crassifolin H (**39**) was obtained from roots of *C. crassifolius* as an angiogenic inhibitor by reducing vessel formation to 59.3% at 15 μg/mL [34]. Tigliane diterpene (**111**) was isolated from the leaves of *C. mauritianus*, which inhibited chikungunya virus-induced cell death in cell culture with EC_50_s of 4.0 ± 0.8 μM [43]. The leaves of *C. tiglium* yielded two tigliane diterpenoids (**135**, **136**), which displayed significant antitubercular activities with MIC values of 19.5, and 20.9 μM, respectively [51]. Compounds (**162**–**165**) were four ent-kaurane diterpenes from *C. tonkinensis*, which significantly stimulated differentiation in osteoblasts [64]. From the twigs and leaves of *C. cascarilloides*, two crotofolane diterpenoid alkaloids cascarinoids B–C (**226**, **227**) were obtained, both of which displayed moderate activities against the ConA-induced proliferation of T lymphocyte cells and/or LPS-induced proliferation of B lymphocyte cells with IC_50_ values ranging from 6.14 to 16.27 μM [71]. Meroditerpenoid (336), from *C. steenkampianus*, showed antiplasmodial activities of 15.8 (D10), 9.1 (W2), and 9.4 (Dd2) μM [102]. Indole alkaloid (**377**) was found in *C. mauritianus* with antioxidant activity (IC_50_, 30.0.0 ± 0.7 μM) by the DPPH radical scavenging assay [117]. Bioactivity-guided fractionation of the root bark of *C. jatrophoides* resulted in the isolation of musidunin (**388**) and musiduol (**389**), both of which showed insect antifeedant activities (PC_50_, 3 μg/mL, PC_95_, 10 μg/mL; PC_50_, 4 μg/mL, PC_95_, 20 μg/mL, respectively) against the second-instar larvae of *Pectinophora gossypiella* in a leaf disk assay [126]. 

## 4. Conclusions

**Figure 14:**
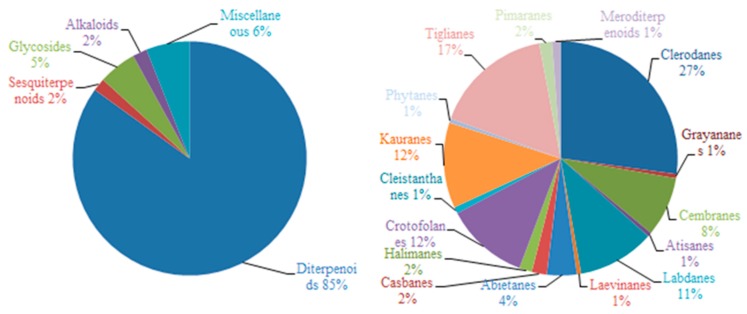
The percentage of each type of compounds (**left**), the percentage of each type of diterpenoids (**right**) from *Croton* Species.

In the present review, we systematically summarized the chemical constituents and biological activity studies of *Croton* species covering from 2006 to 2018. To date, a total of 399 new compounds were reported from *Croton* species, which included 339 diterpenoids, seven sesquiterpenoids, 21 glycosides, eight alkaloids, and 24 miscellaneous compounds (Figure 14). Obviously, diterpenoids are characteristic components for *Croton* species. The diterpenoids with clerodane, tigliane, kaurane, crotofolane, labdane, and cembrane skeletons are among the most studied diterpenoids isolated from *Croton* species (Figure 14). Although the current studies have shown that these isolated compounds from *Croton* species possessed diversified biological activities, many compounds have never been biologically tested. Moreover, most studies conducted so far have focused mainly on in vitro cytotoxic assays. Further studies on the mechanism of actions and the structure activity relationship are needed in order to provide a better understanding of the chemical constituents from *Croton* species as potential medicines. Increasing interest in the chemistry and pharmaceutics of *Croton* species may promote new progress in finding and developing novel compounds.

## Figures and Tables

**Table 1:** Clerodane type diterpenoids from the genus *Croton*.

No.	Compound Name	Molecular Formula	Sources	Ref
**1**	*ent*-3,13*E*-clerodadiene-15-formate	C_21_H_34_O_2_	*C. sylvaticus*	[12]
**2**	9-[2-(2(5*H*)-furanone-4-yl)ethyl]-4,8,9-trimethyl-1,2,3,4,5,6,7,8-octahydronaphthalene-4-carboxylic acid	C_20_H_28_O_4_	*C. crassifolius*	[14]
**3**	9-[2-(2(5*H*)-furanone-4-yl)ethyl]-4,8,9-trimethyl-1,2,3,4,5,6,7,8-octahydronaphthalene-4-carboxylic ester	C_21_H_30_O_4_	*C. crassifolius*	[14]
**4**	Centrafricine I	C_21_H_24_O_6_	*C. mayumbensis*	[19]
**5**	Marrubiagenin	C_20_H_28_O_4_	*C. glabellus*	[15]
**6**	Methyl 15,16-epoxy-3,13(16),14-*ent*-clerodatrien-18,19-olide-17-carboxylate	C_21_H_26_O_5_	*C. oblongifolius*	[29]
**7**	Dimethyl 15,16-epoxy-12-oxo-3,13(16),14-*ent*-clerodatriene-17,18-dicarboxylate	C_22_H_28_O_6_	*C. oblongifolius*	[29]
**8**	Isoteucvin	C_19_H_20_O_5_	*C. jatrophoides*	[30]
**9**	Jatrophoidin	C_21_H_22_O_7_	*C. jatrophoides*	[30]
**10**	8-Epicordatin	C_21_H_26_O_6_	*C. palanostigma*	[31]
**11**	laevigatbenzoate	C_27_H_31_O_5_	*C. laevigatus*	[13]
**12**	3,4,15,16-diepoxy-cleroda-13(16),14-diene-12,17-olide	C_20_H_26_O_4_	*C. oblongifolius*	[22]
**13**	Crassifolin A	C_21_H_30_O_4_	*C. crassifolius*	[16]
**14**	Crassifolin B	C_20_H_29_O_4_	*C. crassifolius*	[16]
**15**	Crassifolin C	C_21_H_24_O_5_	*C. crassifolius*	[16]
**16**	Crassifolin D	C_21_H_24_O_6_	*C. crassifolius*	[16]
**17**	Crassifolin E	C_20_H_23_O_6_	*C. crassifolius*	[16]
**18**	Crassifolin F	C_23_H_29_O_7_	*C. crassifolius*	[16]
**19**	Crassifolin G	C_19_H_20_O_6_	*C. crassifolius*	[16]
**20**	Methyl 3-oxo-12-epibarbascoate	C_21_H_26_O_6_	*C. urucurana*	[32]
**21**	Laevinoids A	C_20_H_22_O_5_	*C. laevigatus*	[20]
**22**	Laevinoids B	C_20_H_23_O_5_Cl	*C. laevigatus*	[20]
**23**	Crotonolide A	C_20_H_18_O_6_	*C. laui*	[21]
**24**	Crotonolide B	C_21_H_24_O_6_	*C. laui*	[21]
**25**	Isocrotonolide B	C_21_H_24_O_6_	*C. laui*	[21]
**26**	Crotonolide C	C_23_H_26_O_8_	*C. laui*	[21]
**27**	Isocrotonolide C	C_23_H_26_O_8_	*C. laui*	[21]
**28**	Crotonolide D	C_21_H_26_O_6_	*C. laui*	[21]
**29**	Isocrotonolide D	C_21_H_26_O_6_	*C. laui*	[21]
**30**	Crotonolide E	C_20_H_26_O_4_	*C. laui*	[21]
**31**	Crotonolide F	C_20_H_26_O_4_	*C. laui*	[21]
**32**	Crotonolide G	C_20_H_32_O	*C. laui*	[21]
**33**	Crotonolide H	C_20_H_32_O_4_	*C. laui*	[21]
**34**	12-Deoxycrotonolide H	C_20_H_32_O_3_	*C. laui*	[21]
**35**	Crotonoligaketone	C_23_H_26_O_8_	*C. oligandrum*	[33]
**36**	Crotonpene A	C_20_H_26_O_3_	*C. yanhuii*	[23]
**37**	Crotonpene B	C_21_H_28_O_5_	*C. yanhuii*	[23]
**38**	Crassifolin I	C_20_H_22_O_6_	*C. crassifolius*	[34]
**39**	Crassifolin H	C_19_H_20_O_5_	*C. crassifolius*	[34]
**40**	Crotoeurin A	C_38_H_36_O_1_	*C. euryphyllus*	[25]
**41**	Crotoeurin B	C_20_H_24_O_6_	*C. euryphyllus*	[25]
**42**	Crotoeurin C	C_20_H_22_O_6_	*C. euryphyllus*	[25]
**43**	3-Oxo-15,16-epoxy-4α,12-dihydroxy-*ent-neo*-clerodan-13(16),14-diene	C_20_H_30_O_4_	*C. limae*	[35]
**44**	15,16-Epoxy-3α,4α,12-trihydroxy-*ent-neo*-clerodan- 13(16),14-diene	C_20_H_32_O_4_	*C. limae*	[35]
**45**	3α,4α,15,16-Tetrahydroxy-*ent-neo*-cleroda-13E-ene	C_20_H_36_O_4_	*C. limae*	[35]
**46**	Cracroson A	C_19_H_21_O_6_	*C. crassifolius*	[26]
**47**	Cracroson B	C_20_H_22_O_6_	*C. crassifolius*	[26]
**48**	Cracroson C	C_19_H_19_O_4_N	*C. crassifolius*	[26]
**49**	Crassifolin J	C_20_H_20_O_5_	*C. crassifolius*	[36]
**60**	Crotocorylifuran-2-one	C_22_H_24_O_8_	*C.megalocarpoides*	[27]
**61**	Megalocarpoidolide D	C_22_H_22_O_8_	*C.megalocarpoides*	[27]
**62**	7,8-Dehydrocrotocorylifuran	C_22_H_24_O_7_	*C.megalocarpoides*	[27]
**63**	Megalocarpoidolide E	C_22_H_24_O_8_	*C.megalocarpoides*	[27]
**64**	Megalocarpoidolide F	C_22_H_24_O_8_	*C.megalocarpoides*	[27]
**65**	Megalocarpoidolide G	C_22_H_24_O_9_	*C.megalocarpoides*	[27]
**66**	Megalocarpoidolide H	C_24_H_28_O_10_	*C.megalocarpoides*	[27]
**67**	Launine K	C_27_H_36_O_3_	*C. laui*	[37]
**68**	Crassin A	C_17_H_20_O_4_	*C. crassifolius*	[17]
**69**	Crassin B	C_17_H_20_O_4_	*C. crassifolius*	[17]
**70**	Crassin C	C_21_H_24_O_6_	*C. crassifolius*	[17]
**71**	Crassin D	C_20_H_20_O_5_	*C. crassifolius*	[17]
**72**	Crassin E	C_19_H_20_O_3_	*C. crassifolius*	[17]
**73**	Crassin F	C_19_H_18_O_7_	*C. crassifolius*	[17]
**74**	Crassin G	C_20_H_26_O_5_	*C. crassifolius*	[17]
**75**	Crassin H	C_21_H_30_O_5_	*C. crassifolius*	[17]
**76**	Crassifolius A	C_20_H_22_O_5_	*C. crassifolius*	[38]
**77**	Crassifolius B	C_21_H_24_O_6_	*C. crassifolius*	[38]
**78**	Crassifolius C	C_21_H_26_O_5_	*C. crassifolius*	[38]
**79**	Crolaevinoid C	C_27_H_28_O_6_	*C. laevigatus*	[39]
**80**	Crolaevinoid D	C_27_H_32_O_8_	*C. laevigatus*	[39]
**81**	Crolaevinoid E	C_20_H_28_O_6_	*C. laevigatus*	[39]
**82**	Crolaevinoid F	C_21_H_30_O_5_	*C. laevigatus*	[39]
**83**	Norcrassifolin	C_19_H_18_O_4_	*C. crassifolius*	[28]
**84**	Hypolein A	C_20_H_26_O_4_	*C. hypoleucus*	[24]
**85**	Hypolein B	C_20_H_28_O_3_	*C. hypoleucus*	[24]
**86**	Hypolein C	C_20_H_28_O_3_	*C. hypoleucus*	[24]
**87**	Cracroson E	C_19_H_20_O_6_	*C. crassifolius*	[40]
**88**	Cracroson F	C_19_H_20_O_6_	*C. crassifolius*	[40]
**89**	Cracroson G	C_21_H_26_O_7_	*C. crassifolius*	[40]
**90**	12-Epi-megalocarpoidolide D	C_22_H_22_O_8_	*C. oligandrus*	[18]
**91**	Crotonolins A	C_22_H_22_O_10_	*C. oligandrus*	[18]
**92**	Crotonolins B	C_22_H_22_O_10_	*C. oligandrus*	[18]

**Table 2:** Tigliane type diterpenoids from the genus *Croton*.

No.	Compound Name	Molecular Formula	Sources	Ref
**93**	12-*O*-isobutyrylphorbol-13-decanoate	C_34_H_52_O_8_	*C. tiglium*	[45]
**94**	12-*O*-(2-methyl)butyrylphorbol-13-octanoate	C_33_H_50_O_8_	*C. tiglium*	[45]
**95**	12-*O*-[(2R)-*N*,*N*-dimethyl-3-methylbutanoyl]-4-deoxyphorbol 13-acetate	C_29_H_43_NO_7_	*C. ciliatoglandulifer*	[41]
**96**	12-*O*-[(2*S*)-*N*,*N*-dimethyl-3-methylbutanoyl]-4-deoxyphorbol 13-acetate	C_29_H_43_NO_7_	*C. ciliatoglandulifer*	[41]
**97**	12-*O*-[(2*R*)-*N*,*N*-Dimethyl-3-methylbutanoyl]phorbol 13-acetate	C_29_H_43_NO_8_	*C. ciliatoglandulifer*	[41]
**98**	12-*O*-[3-Methyl-2-butenoyl]-4-deoxyphorbol 13-acetate	C_27_H_36_NO_7_	*C. ciliatoglandulifer*	[41]
**99**	12-*O*-(2-methyl)butyrylphorbol-13-tiglate	C_30_H_42_O_8_	*C. tiglium*	[46]
**100**	12-*O*-tiglylphorbol-13-propionate	C_28_H_38_O_8_	*C. tiglium*	[46]
**101**	13-*O*-acetylphorbol-20-oleate	C_40_H_62_O_8_	*C. tiglium*	[46]
**106**	12-*O*-tiglyl-4-deoxy-4*α*-phorbol-13-(2-methyl)butyrate	C_30_H_42_O_7_	*C. tiglium*	[46]
**107**	Alienusolin	C_42_H_66_O_8_	*C. alienus*	[42]
**108**	12-*O*-acetyl-5,6-didehydro-7-oxophorbol-13-yl 2-methylbutanoate	C_27_H_36_O_9_	*C. tiglium*	[47]
**109**	12-*O*-acetyl-5,6-didehydro-7-oxophorbol-13-yl2-methylpropanoate	C_26_H_34_O_9_	*C. tiglium*	[47]
**110**	12-Oacetyl-5,6-didehydro-6,7-dihydro-7-hydroxyphorbol-13-yl 2-methylbutanoate	C_27_H_38_O_9_	*C. tiglium*	[47]
**111**	12-*O*-decanoyl-7-hydroperoxy-phorbol-5-ene-13-acetate	C_32_H_42_O_10_	*C. mauritianus*	[43]
**112**	20-deoxy-20-oxophorbol12-tiglate 13-(2-methyl)butyrate	C_30_H_40_O_8_	*C. tiglium*	[48]
**113**	12-*O*-acetylphorbol-13-isobutyrate	C_26_H_36_O_8_	*C. tiglium*	[48]
**114**	12-*O*-benzoylphorbol-13-(2-methyl)butyrate	C_32_H_40_O_8_	*C. tiglium*	[48]
**115**	12-*O*-tiglyl-7-oxo-5-ene-phorbol-13-(2-methyl)butyrate	C_30_H_40_O_9_	*C. tiglium*	[48]
**116**	13-*O*-(2-metyl)butyryl-4-deoxy-4a-phorbol	C_25_H_36_O_6_	*C. tiglium*	[48]
**117**	Crotignoid A	C_30_H_42_O_10_	*C. tiglium*	[49]
**118**	Crotignoid B	C_29_H_40_O_10_	*C. tiglium*	[49]
**119**	Crotignoid C	C_30_H_42_O_9_	*C. tiglium*	[49]
**120**	Crotignoid D	C_29_H_40_O_9_	*C. tiglium*	[49]
**121**	Crotignoid E	C_29_H_38_O_9_	*C. tiglium*	[49]
**122**	Crotignoid F	C_28_H_36_O_9_	*C. tiglium*	[49]
**123**	Crotignoid G	C_30_H_44_O_8_	*C. tiglium*	[49]
**124**	Crotignoid H	C_29_H_38_O_8_	*C. tiglium*	[49]
**125**	Crotignoid I	C_30_H_44_O_8_	*C. tiglium*	[49]
**126**	Crotignoid J	C_31_H_38_O_8_	*C. tiglium*	[49]
**127**	Crotignoid K	C_29_H_34_O_7_	*C. tiglium*	[49]
**128**	Crotusin A	C_36_H_54_O_10_	*C. caudatus*	[44]
**129**	Crotusin B	C_46_H_72_O_11_	*C. caudatus*	[44]
**130**	Crotusin C	C_36_H_52_O_11_	*C. caudatus*	[44]
**131**	12-*O*-tiglylphorbol-4-deoxy- 4*β*-phorbol-13-acetate	C_27_H_36_O_7_	*C. tiglium*	[50]
**132**	12-*O*-tiglylphorbol-4-deoxy-4*β*-phorbol-13-hexadecanoate	C_41_H_64_O_7_	*C. tiglium*	[50]
**133**	13-*O*-acetylphorbol-4-deoxy-4*β*-phorbol-20-oleate	C_40_H_62_O_7_	*C. tiglium*	[50]
**134**	13-*O*-acetylphorbol-4-deoxy-4*β*-phorbol-20-linoleate	C_40_H_60_O_7_	*C. tiglium*	[50]
**135**	4-deoxy-20-oxophorbol 12-tiglyl 13-acetate	C_27_H_34_O_7_	*C. tiglium*	[51]
**136**	7-oxo-5-ene-phorbol-13-(2-methylbutyrate)	C_25_H_34_O_8_	*C. tiglium*	[51]
**137**	7-hydroxyl-phorbol-5-ene-13-(2-methyl)butyrate	C_25_H_36_O_8_	*C. tiglium*	[51]

**Table 3:** Kaurane type diterpenoids from the genus *Croton*.

No.	Compound Name	Molecular Formula	Sources	Ref
**149**	Caracasine	C_21_H_30_O_3_	*C. caracasana*	[53]
**150**	Caracasine acid	C_20_H_28_O_3_	*C. caracasana*	[53]
**151**	Kongensin A	C_22_H_30_O_5_	*C. kongensis*	[56]
**152**	Kongensin B	C_22_H_30_O_6_	*C. kongensis*	[56]
**153**	Kongensin C	C_20_H_28_O_5_	*C. kongensis*	[56]
**154**	Kongensin D	C_20_H_28_O_4_	*C. kongensis*	[57]
**155**	Kongensin E	C_26_H_36_O_7_	*C. kongensis*	[57]
**156**	Kongensin F	C_24_H_34_O_5_	*C. kongensis*	[58]
**157**	Crotonkinin A	C_20_H_30_O_2_	*C. tonkinensis*	[62]
**158**	Crotonkinin B	C_22_H_32_O_4_	*C. tonkinensis*	[62]
**159**	14-*epi*-hyalic acid	C_20_H_28_O_4_	*C. argyrophylloides*	[63]
**160**	14-[(2-methylbutanoyl)oxy]-3,4-seco-*ent*-kaura-4(19),16-dien-3-oic acid	C_25_H_39_O_4_	*C. megistocarpus*	[54]
**161**	14-{[(2*Z*)-2-methylbut-2-enoyl]oxy}-3,4-seco-*ent*-kaura-4(19),16-dien-3-oic acid	C_25_H_37_O_4_	*C. megistocarpus*	[54]
**162**	*ent*-11*β*-acetoxykaur-16-en-18-ol	C_22_H_34_O_3_	*C. tonkinensis*	[64]
**163**	*ent*-11*α*-hydroxy-18-acetoxykaur-16-ene	C_22_H_34_O_3_	*C. tonkinensis*	[64]
**164**	*ent*-14*β*-hydroxy-18-acetoxykaur-16-ene	C_22_H_34_O_3_	*C. tonkinensis*	[64]
**165**	*ent*-7*α*-hydroxy-18-acetoxykaur-16-ene	C_22_H_34_O_3_	*C. tonkinensis*	[64]
**166**	*ent*-14S*-hydroxykaur-16-en-19-oic acid	C_20_H_30_O_3_	*C. pseudopulchellus*	[65]
**167**	*ent*-14S*,17-dihydroxykaur-15-en-19-oic acid	C_20_H_30_O_4_	*C. pseudopulchellus*	[65]
**168**	*ent*-3,4-*seco*-17-oxo-kaur-4(19),15(16)-dien-3-oic acid	C_20_H_28_O_3_	*C. oblongifolius*	[55]
**169**	Crotonkinin C	C_22_H_30_O_5_	*C. tonkinensis*	[66]
**170**	Crotonkinin D	C_24_H_34_O_6_	*C. tonkinensis*	[66]
**171**	Crotonkinin E	C_24_H_34_O_5_	*C. tonkinensis*	[66]
**172**	Crotonkinin F	C_24_H_34_O_5_	*C. tonkinensis*	[66]
**173**	Crotonkinin G	C_23_H_36_O_5_	*C. tonkinensis*	[66]
**174**	Crotonkinin H	C_22_H_36_O_4_	*C. tonkinensis*	[66]
**175**	Crotonkinin I	C_24_H_36_O_5_	*C. tonkinensis*	[66]
**176**	Crotonkinin J	C_23_H_34_O_5_	*C. tonkinensis*	[66]
**177**	14*β*-hydroxy-3-oxo-ent-kaur-16-ene	C_20_H_30_O_2_	*C. kongensis*	[67]
**178**	Kongeniod A	C_21_H_30_O_3_	*C. kongensis*	[59]
**179**	Kongeniod B	C_21_H_30_O_4_	*C. kongensis*	[59]
**180**	Kongeniod C	C_23_H_32_O_5_	*C. kongensis*	[59]
**181**	15-oxo-17(10′-*α*-pinenyl)-kauran-18-oic acid	C_30_H_44_O_3_	*C. limae*	[35]
**182**	Micansinoic acid	C_40_H_58_O_7_	*C. micans*	[60]
**183**	Isomicansinoic acid	C_40_H_58_O_7_	*C. micans*	[60]
**184**	Dimethylester of micansinoic	C_42_H_62_O_7_	*C. micans*	[60]
**185**	Methyl-micansinoic acid	C_41_H_60_O_7_	*C. micans*	[60]
**186**	Ethyl-micansinoic acid	C_42_H_62_O_7_	*C. micans*	[60]
**187**	Crotonkinensin C	C_40_H_62_O_8_	*C. tonkinensis*	[61]
**188**	Crotonkinensin D	C_44_H_66_O_10_	*C. tonkinensis*	[61]

**Table 4:** Crotofolane type diterpenoids from the genus *Croton*.

No.	Compound Name	Molecular Formula	Sources	Ref
**189**	Crotocascarin A	C_25_H_32_O_7_	*C. cascarilloides*	[68]
**190**	Crotocascarin B	C_25_H_32_O_7_	*C. cascarilloides*	[68]
**191**	Crotocascarin C	C_25_H_32_O_8_	*C. cascarilloides*	[68]
**192**	Crotocascarin D	C_25_H_32_O_6_	*C. cascarilloides*	[68]
**193**	Crotocascarin E	C_26_H_34_O_8_	*C. cascarilloides*	[68]
**194**	Crotocascarin F	C_24_H_30_O_7_	*C. cascarilloides*	[68]
**195**	Crotocascarin G	C_24_H_30_O_7_	*C. cascarilloides*	[68]
**196**	Crotocascarin H	C_24_H_30_O_8_	*C. cascarilloides*	[68]
**197**	Crotocascarin *α*	C_24_H_32_O_8_	*C. cascarilloides*	[68]
**198**	Crotocascarin *β*	C_24_H_32_O_7_	*C. cascarilloides*	[68]
**199**	(5*β*,6*β*)-5,6: 13,16-diepoxycrotofola-4(9),10(18),13,15-tetraen-1-one	C_20_H_22_O_3_	*C. argyrophyllus*	[72]
**200**	(5*β*,6*β*)-5,6: 13,16-diepoxy-2-epicrotofola-4(9),10(18),13,15-tetraen-1-one	C_20_H_22_O_3_	*C. argyrophyllus*	[72]
**201**	(5*β*,6*β*)-5,6: 13,16-diepoxy-16-hydroxycrotofola-4(9),10(18),13,15-tetraen-1-one	C_20_H_22_O_4_	*C. argyrophyllus*	[72]
**202**	(5*β*,6*β*)-5,6: 13,16-diepoxy-16-hydroxy-2-epi-crotofola-4(9),10(18),13,15-tetraen-1-one	C_20_H_22_O_4_	*C. argyrophyllus*	[72]
**203**	Crotocarasin A	C_20_H_22_O_4_	*C. caracasanus*	[73]
**204**	Crotocarasin B	C_20_H_22_O_4_	*C. caracasanus*	[73]
**205**	Crotocarasin C	C_22_H_26_O_5_	*C. caracasanus*	[73]
**206**	Crotocarasin D	C_22_H_26_O_5_	*C. caracasanus*	[73]
**207**	EBC-162	C_20_H_24_O_2_	*C. insularis*	[74]
**208**	EBC-233	C_20_H_24_O_4_	*C. insularis*	[74]
**209**	EBC-300	C_20_H_24_O_4_	*C. insularis*	[74]
**210**	EBC-240	C_20_H_26_O_5_	*C. insularis*	[74]
**211**	EBC-241	C_20_H_26_O_5_	*C. insularis*	[74]
**212**	Crotocascarin I	C_20_H_24_O_5_	*C. cascarilloides*	[69]
**213**	Crotocascarin J	C_20_H_24_O_6_	*C. cascarilloides*	[69]
**214**	Crotocascarin K	C_20_H_24_O_5_	*C. cascarilloides*	[69]
**215**	Crotocascarin *γ*	C_19_H_24_O_6_	*C. cascarilloides*	[69]
**216**	Crotocascarin L	C_22_H_26_O_7_	*C. cascarilloides*	[70]
**217**	Crotocascarin M	C_21_H_26_O_6_	*C. cascarilloides*	[70]
**218**	Crotocascarin N	C_20_H_22_O_6_	*C. cascarilloides*	[70]
**219**	Crotocascarin O	C_25_H_34_O_9_	*C. cascarilloides*	[70]
**220**	Crotocascarin P	C_25_H_34_O_8_	*C. cascarilloides*	[70]
**221**	Crotocascarin Q	C_25_H_32_O_7_	*C. cascarilloides*	[70]
**222**	Neocrotocascarin	C_25_H_32_O_8_	*C. cascarilloides*	[70]
**223**	Crotodichogamoin A	C_20_H_22_O_4_	*C. dichogamus*	[75]
**224**	Crotodichogamoin B	C_20_H_22_O_2_	*C. dichogamus*	[75]
**225**	Cascarinoid A	C_28_H_31_NO_5_	*C. cascarilloides*	[71]
**226**	Cascarinoid B	C_28_H_31_NO_5_	*C. cascarilloides*	[71]
**227**	Cascarinoid C	C_28_H_31_NO_6_	*C. cascarilloides*	[71]

**Table 5:** Labdane type diterpenoids from the genus *Croton*.

No.	Compound Name	Molecular Formula	Sources	Ref
**228**	Labdinine N	C_20_H_34_O_3_	*C. laui*	[76]
**229**	*ent*-12,15-dioxo-3,4-*seco*-4,8,13-labdatrien-3-oic acid	C_20_H_28_O_4_	*C. stipuliformis*	[78]
**230**	*ent*-12,15-epoxy-3,4-*seco*-4,8,12,14-labdatetraen-3-oic acid	C_20_H_28_O_3_	*C. stipuliformis*	[78]
**231**	*ent*-15-nor-14-oxo-3,4-seco-4,8,12(*E*)-labdatrien-3-oic acid	C_19_H_28_O_3_	*C. stipuliformis*	[78]
**232**	*ent*-12,15-dioxo-8,13-labdadien-3*a*-ol	C_20_H_28_O_3_	*C. stipuliformis*	[78]
**233**	Crotonlaevin A	C_18_H_30_O_4_	*C. laevigatus*	[79]
**234**	Crotonlaevin B	C_20_H_32_O_5_	*C. laevigatus*	[79]
**235**	Crotonlaevin C	C_21_H_34_O_5_	*C. laevigatus*	[79]
**236**	Crotonlaevin D	C_18_H_30_O_3_	*C. laevigatus*	[79]
**237**	Crotonlaevin E	C_20_H_32_O_5_	*C. laevigatus*	[79]
**238**	Crotonlaevin F	C_22_H_34_O_6_	*C. laevigatus*	[79]
**239**	Crotonlaevin G	C_22_H_36_O_5_	*C. laevigatus*	[79]
**240**	Crotonlaevin H	C_22_H_36_O_5_	*C. laevigatus*	[79]
**241**	Crotonlaevin I	C_20_H_34_O_4_	*C. laevigatus*	[79]
**242**	Crotonlaevin J	C_20_H_30_O_3_	*C. laevigatus*	[79]
**243**	Crotonlaevin K	C_20_H_28_O_3_	*C. laevigatus*	[79]
**244**	Crotonlaevin L	C_20_H_30_O_4_	*C. laevigatus*	[79]
**245**	Crotonlaevin M	C_20_H_30_O_4_	*C. laevigatus*	[79]
**246**	Crotonlaevin N	C_20_H_30_O_3_	*C. laevigatus*	[79]
**247**	Crotonlaevin O	C_20_H_30_O_3_	*C. laevigatus*	[79]
**248**	Crotonlaevin P	C_20_H_30_O_3_	*C. laevigatus*	[79]
**249**	Crotonolide I	C_20_H_34_O_3_	*C. laui*	[21]
**250**	Crotonolide J	C_19_H_30_O_3_	*C. laui*	[21]
**251**	Launine A	C_19_H_32_O_3_	*C. laui*	[82]
**252**	Launine B	C_19_H_32_O_4_	*C. laui*	[82]
**253**	Launine C	C_20_H_34_O_3_	*C. laui*	[82]
**254**	Launine D	C_20_H_34_O_3_	*C. laui*	[82]
**255**	Launine E	C_20_H_32_O_5_	*C. laui*	[82]
**256**	Launine F	C_20_H_32_O_5_	*C. laui*	[82]
**257**	Launine G	C_20_H_30_O_4_	*C. laui*	[82]
**258**	Launine H	C_20_H_30_O_4_	*C. laui*	[82]
**259**	Launine I	C_20_H_34_O_3_	*C. laui*	[82]
**260**	15,16-epoxy-4-hydroxy-labda-13(16),14-dien-3,12-dione	C_20_H_28_O_4_	*C. jacobinensis*	[77]
**261**	Crotondecalvatin A	C_29_H_42_O_4_	*C. decalvatus*	[80]
**262**	Crotondecalvatin B	C_30_H_42_O_6_	*C. decalvatus*	[80]
**263**	Bicrotonol A	C_40_H_68_O_4_	*C. crassifolius*	[81]

**Table 6:** Cembrane type diterpenoids from the genus *Croton*.

No.	Compound Name	Molecular Formula	Sources	Ref
**264**	Launine O	C_20_H_34_O_2_	*C. laui*	[76]
**265**	Launine P	C_21_H_36_O_2_	*C. laui*	[76]
**266**	Furanocembranoid 1	C_20_H_30_O_2_	*C. oblongifolius*	[83]
**267**	Furanocembranoid 2	C_20_H_30_O_3_	*C. oblongifolius*	[83]
**268**	Furanocembranoid 3	C_20_H_32_O_4_	*C. oblongifolius*	[83]
**269**	Furanocembranoid 4	C_20_H_32_O_5_	*C. oblongifolius*	[83]
**270**	Laevigatlactone A	C_20_H_30_O_3_	*C. laeVigatus*	[84]
**271**	Laevigatlactone C	C_20_H_30_O_3_	*C. laeVigatus*	[84]
**272**	Laevigatlactone B	C_20_H_30_O_3_	*C. laeVigatus*	[84]
**273**	Laevigatlactone D	C_20_H_30_O_3_	*C. laeVigatus*	[84]
**274**	Laevigatlactone E	C_20_H_30_O_4_	*C. laeVigatus*	[84]
**275**	Laevigatlactone F	C_20_H_30_O_5_	*C. laeVigatus*	[84]
**276**	(+)-[1R*,2S*,7S*,8S*,12R*]-7,8-Epoxy-2,12-cyclocembra-3*E*,10*Z*dien-20,10-olide	C_20_H_28_O_3_	*C. gratissimus*	[85]
**277**	(+)-[1R*,10R*]-Cembra-2*E*,4*E*,7*E*,11*Z*-tetraen-20,10-olide	C_20_H_28_O_2_	*C. gratissimus*	[85]
**278**	(+)-[1R*,4S*,10R*]-4-Hydroxycembra-2*E*,7*E*,11*Z*-trien-20,10-olide	C_20_H_30_O_3_	*C. gratissimus*	[85]
**279**	(−)-[1R*,4R*,10R*]-4-Hydroxycembra-2*E*,7*E*,11*Z*-trien-20,10-olide	C_20_H_30_O_3_	*C. gratissimus*	[85]
**280**	(−)-(1R*,4R*,10R*)-4-Methoxycembra-2*E*,7*E*,11*Z*-trien-20,10-olide	C_21_H_32_O_3_	*C. gratissimus*	[86]
**281**	(−)-(1S*,4R*,10R*)-1-Hydroxy-4-methoxycembra-2*E*,7*E*,11*Z*trien-20,10-olide	C_21_H_32_O_4_	*C. gratissimus*	[86]
**282**	(−)-(1S*,4S*,10R*)-1,4-Dihydroxycembra-2*E*,7*E*,11*Z*-trien-20,10-olide	C_20_H_30_O_4_	*C. gratissimus*	[86]
**283**	(−)-(1S*,4S*,10R*)-1,4-Dihydroxycembra-2*E*,7*E*,11*Z*-trien-20,10-olide	C_20_H_30_O_4_	*C. gratissimus*	[86]
**284**	(+)-(10R*)-Cembra-1*E*,3*E*,7*E*,11*Z*,16-pentaen-20,10-olide	C_20_H_26_O	*C. gratissimus*	[86]
**285**	(+)-(10R*)-Cembra-1*Z*,3*Z*,7*E*,11*Z*,15-pentaen-20,10-olide	C_20_H_26_O	*C. gratissimus*	[86]
**286**	(+)-(5R*,10R*)-5-Methoxycembra-1*E*,3*E*,7*E*,11*Z*,15-pentaen-20,10-olide	C_21_H_30_O_3_	*C. gratissimus*	[86]
**287**	(+)-(1S*,4S*,7R*,10R*)-1,4,7-Trihydroxycembra-2*E*,8(19),11*Z*-trien-20,10-olide	C_20_H_30_O_5_	*C. gratissimus*	[86]
**288**	(−)-(1S*,4S*,7S*,10R*)-1,4,7-Trihydroxycembra-2*E*,8(19),11*Z*-trien-20,10-olide	C_20_H_30_O_3_	*C. gratissimus*	[86]
**289**	(+)-(1S*,4R*,8S*,10R*)-1,4,8-Trihydroxycembra-2*E*,6*E*,11*Z*-trien-20,10-olide	C_20_H_30_O_5_	*C. gratissimus*	[86]
**290**	Cembranoid 1	C_20_H_30_O_4_	*C. longissimus*	[87]
**291**	Cembranoid 2	C_20_H_30_O_3_	*C. longissimus*	[87]
**281**	(−)-(1S*,4R*,10R*)-1-Hydroxy-4-methoxycembra-2*E*,7*E*,11*Z*trien-20,10-olide	C_21_H_32_O_4_	*C. gratissimus*	[86]
**282**	(−)-(1S*,4S*,10R*)-1,4-Dihydroxycembra-2*E*,7*E*,11*Z*-trien-20,10-olide	C_20_H_30_O_4_	*C. gratissimus*	[86]
**283**	(−)-(1S*,4S*,10R*)-1,4-Dihydroxycembra-2*E*,7*E*,11*Z*-trien-20,10-olide	C_20_H_30_O_4_	*C. gratissimus*	[86]
**284**	(+)-(10R*)-Cembra-1*E*,3*E*,7*E*,11*Z*,16-pentaen-20,10-olide	C_20_H_26_O	*C. gratissimus*	[86]
**285**	(+)-(10R*)-Cembra-1*Z*,3*Z*,7*E*,11*Z*,15-pentaen-20,10-olide	C_20_H_26_O	*C. gratissimus*	[86]
**286**	(+)-(5R*,10R*)-5-Methoxycembra-1*E*,3*E*,7*E*,11*Z*,15-pentaen-20,10-olide	C_21_H_30_O_3_	*C. gratissimus*	[86]
**287**	(+)-(1S*,4S*,7R*,10R*)-1,4,7-Trihydroxycembra-2*E*,8(19),11*Z*-trien-20,10-olide	C_20_H_30_O_5_	*C. gratissimus*	[86]
**288**	(−)-(1S*,4S*,7S*,10R*)-1,4,7-Trihydroxycembra-2*E*,8(19),11*Z*-trien-20,10-olide	C_20_H_30_O_3_	*C. gratissimus*	[86]
**289**	(+)-(1S*,4R*,8S*,10R*)-1,4,8-Trihydroxycembra-2*E*,6*E*,11*Z*-trien-20,10-olide	C_20_H_30_O_5_	*C. gratissimus*	[86]
**290**	Cembranoid 1	C_20_H_30_O_4_	*C. longissimus*	[87]
**291**	Cembranoid 2	C_20_H_30_O_3_	*C. longissimus*	[87]

**Table 7:** Abietane type diterpenoids from the genus *Croton*.

No.	Compound Name	Molecular Formula	Sources	Ref
**292**	Isolophanthin E	C_20_H_30_O_3_	*C. megalocarpoides*	[27]
**293**	*rel*-(1*R*,4a*R*,5*R*,8*R*)-methyl-7-(1-(methoxycarbonyl)vinyl)-5,8-diacetoxy-1,2,3,4*a*,5,6,7,8,9,10,10*a*-dodecahydro-1,4*a*-dimethyl-2-oxophenanthrene-1-carboxylate	C_26_H_34_O_9_	*C. argyrophylloides*	[63]
**294**	Crotontomentosin A	C_20_H_26_O_2_	*C. caudatus*	[88]
**295**	Crotontomentosin B	C_20_H_30_O_3_	*C. caudatus*	[88]
**296**	Crotontomentosin D	C_20_H_24_O_2_	*C. caudatus*	[88]
**297**	Crotontomentosin C	C_20_H_28_O_2_	*C. caudatus*	[88]
**298**	Crotontomentosin E	C_22_H_32_O_3_	*C. caudatus*	[88]
**299**	Crotolaevigatone A	C_20_H_24_O_3_	*C. laevigatus*	[89]
**300**	Crotolaevigatone B	C_20_H_26_O_2_	*C. laevigatus*	[89]
**301**	Crotolaevigatone C	C_20_H_26_O_3_	*C. laevigatus*	[89]
**302**	Crotolaevigatone D	C_20_H_28_O_4_	*C. laevigatus*	[89]
**303**	Crotolaevigatone E	C_19_H_24_O_2_	*C. laevigatus*	[89]
**304**	Crotolaevigatone F	C_20_H_30_O_4_	*C. laevigatus*	[89]
**305**	Crotolaevigatone G	C_20_H_30_O_4_	*C. laevigatus*	[89]

**Table 8:** Casbane type diterpenoids from the genus *Croton*.

No.	Compound Name	Molecular Formula	Sources	Ref
**306**	1,4-dihydroxy-2*E*,6*E*,12*E*-trien-5-one-casbane	C_20_H_30_O_3_	*C.* *nepetaefolius*	[90]
**307**	4-hydroxy-2*E*,6*E*,12*E*-5-one-casbane	C_20_H_29_O_3_	*C.* *nepetaefolius*	[90]
**308**	1-hydroxy-(2*E*,6*Z*,12*E*)-casba-2,6,12-triene-4,5-dione	C_20_H_28_O_3_	*C.* *argyrophyllus*	[91]
**309**	6*E*,12*E*-casba-1,3,6,12-tetraen-1,4-epoxy-5-one	C_20_H_26_O_2_	*C.* *argyrophyllus*	[91]
**310**	(2*E*,5*β*,6*E*,12*E*)-5-hydroxycasba-2,6,12-trien-4-one	C_20_H_30_O_2_	*C. argyrophyllus*	[72]
**311**	EBC-324	C_20_H_28_O_5_	*C. insularis*	[92]
**312**	EBC-329	C_20_H_26_O_4_	*C. insularis*	[92]

**Table 9:** Halimane type diterpenoids from the genus *Croton*.

No.	Compound Name	Molecular Formula	Sources	Ref
**313**	Crassifoliusin A	C_21_H_24_O_5_	*C.* *crassifolius*	[95]
**314**	Crotontomentosin F	C_21_H_30_O_3_	*C.* *caudatus*	[88]
**315**	Crolaevinoid A	C_27_H_30_O_7_	*C.* *laevigatus*	[39]
**316**	Crolaevinoid B	C_20_H_26_O_4_	*C.* *laevigatus*	[39]
**317**	Crothalimene A	C_20_H_26_O_4_	*C.* *dichogamus*	[75]
**318**	Crothalimene B	C_20_H_30_O_2_	*C.* *dichogamus*	[75]

**Table 10:** Pimarane type diterpenoids from the genus *Croton*.

No.	Compound Name	Molecular Formula	Sources	Ref
**319**	*ent*-3*β*-hydroxypimara-8(14),9,15-trien-12-one	C_20_H_28_O_2_	*C.* *insularis*	[98]
**320**	EBC-316	C_20_H_26_O_2_	*C.* *insularis*	[99]
**321**	EBC-325	C_20_H_26_O_4_	*C.* *insularis*	[99]
**322**	EBC-326	C_20_H_26_O_4_	*C.* *insularis*	[99]
**323**	EBC-327	C_20_H_24_O_3_	*C.* *insularis*	[99]
**324**	EBC-345	C_20_H_30_O_4_	*C.* *insularis*	[99]

**Table 11:** Cleistanthane type diterpenoids from the genus *Croton*.

No.	Compound Name	Molecular Formula	Sources	Ref
**325**	3-hydroxycleistantha-13(17),15-diene	C_20_H_32_O	*C.* *oblongifolius*	[93]
**326**	3,4-*seco*-cleistantha-4(18),13(17),15-trien-3-oic acid	C_20_H_30_O_2_	*C.* *oblongifolius*	[93]
**327**	*rel*-(5*β*,8*α*,10*α*)-8-hydroxy-13-methylpodocarpa-9(11),13-diene-3,12-dione	C_18_H_25_O_3_	*C.* *regelianus*	[94]

**Table 12:** Grayanane type diterpenoids from the genus *Croton*.

No.	Compound Name	Molecular Formula	Sources	Ref
**328**	Crotonkinensin A	C_20_H_24_O_4_	*C.* *tonkinensis*	[100]
**329**	Crotonkinensin B	C_20_H_26_O_3_	*C.* *tonkinensis*	[100]

**Table 13:** Atisane type diterpenoids from the genus *Croton*.

No.	Compound Name	Molecular Formula	Sources	Ref
**330**	Crotobarin	C_22_H_28_O_5_	*C.* *barorum*	[101]
**331**	Crotogoudin	C_20_H_26_O_3_	*C.* *goudotii*	[101]

**Table 14:** Phytane type diterpenoids from the genus *Croton*.

No.	Compound Name	Molecular Formula	Sources	Ref
**332**	Launine L	C_20_H_32_O_3_	*C.* *laui*	[37]
**333**	Launine M	C_20_H_32_O_2_	*C.* *laui*	[37]

**Table 15:** Laevinane type diterpenoids from the genus *Croton*.

No.	Compound Name	Molecular Formula	Sources	Ref
**334**	Crolaevinoid G	C_20_H_24_O_6_	*C.* *laevigatus*	[39]
**335**	Crolaevinoid H	C_21_H_26_O_6_	*C.* *laevigatus*	[39]

**Table 16:** Meroditerpenoids from the genus *Croton*.

No.	Compound Name	Molecular Formula	Sources	Ref
**336**	Steenkrotin A	C_20_H_24_O_6_	*C.* *steenkampianus*	[102]
**337**	Steenkrotin B	C_20_H_28_O_7_	*C.* *steenkampianus*	[102]
**338**	Norcrassin A	C_17_H_22_O_7_	*C.* *crassifolius*	[81]
**339**	Cracroson D	C_21_H_26_O_6_	*C.* *crassifolius*	[40]

**Table 17:** Sesquiterpenoids from the genus *Croton*.

No.	Compound Name	Molecular Formula	Sources	Ref
**340**	6*α* -methoxy-cyperene	C_16_H_26_O	*C.* *muscicarpa*	[103]
**341**	*rel*-(1*R*,4*S*,6*R*,7*S*,8*αR*)-decahydro-1-(hydroxymethyl)-4,9,9-trimethyl-4,7-(epoxymethano)azulen-6-ol	C_15_H_26_O_3_	*C. regelianus*	[94]
**342**	Blumenol A	C_13_H_20_O_3_	*C. pedicellatus*	[104]
**343**	Crocrassins A	C_15_H_24_O_3_	*C. crassifolius*	[105]
**344**	Crocrassins B	C_16_H_26_O_3_	*C. crassifolius*	[105]
**345**	1,3,5-cadinatriene-(7*R*,10*S*)-diol	C_15_H_25_O_2_	*C. dichogamus*	[75]
**346**	Cracroson H	C_15_H_22_O_3_	*C. crassifolius*	[40]

**Table 18:** Sesterterpenoid from the genus *Croton*.

No.	Compound Name	Molecular Formula	Sources	Ref
**347**	Pseudopulchellol	C_25_H_40_O	*C.pseudopulchellus*	[106]

**Table 19:** Triterpenoid from the genus *Croton*.

No.	Compound Name	Molecular Formula	Sources	Ref
**348**	3*α*-hydroxy-urs-12,15-dien	C_30_H_48_O	*C.* *bonplandianum*	[107]

**Table 20:** Glycosides from the genus *Croton*.

No.	Compound Name	Molecular Formula	Sources	Ref
**349**	Cyperenoic acid-9-*O*-*β*-d-glucopyranoside	C_21_H_32_O_8_	*C. crassifolius*	[108]
**350**	3-*O*-*β*-d-xylopyranosylspathodic acid	C_35_H_56_O_9_	*C. lachnocarpus*	[109]
**351**	Helichrysoside-3*′*-methylether	C_31_H_28_O_14_	*C. zambesicus*	[110]
**352**	Crotonionoside A	C_29_H_42_O_11_	*C. cascarilloides*	[111]
**353**	Crotonionoside B	C_30_H_44_O_12_	*C. cascarilloides*	[111]
**354**	Crotonionoside C	C_24_H_42_O_12_	*C. cascarilloides*	[111]
**355**	Crotonionoside D	C_31_H_46_O_14_	*C. cascarilloides*	[111]
**356**	Crotonionoside E	C_35_H_52_O_16_	*C. cascarilloides*	[111]
**357**	Crotonionoside F	C_24_H_42_O_11_	*C. cascarilloides*	[111]
**358**	Crotonionoside G	C_29_H_40_O_11_	*C. cascarilloides*	[111]
**359**	Oblongionoside A	C_24_H_42_O_12_	*C. oblongifolius*	[112]
**360**	Oblongionoside B	C_24_H_42_O_12_	*C. oblongifolius*	[112]
**361**	Oblongionoside C	C_24_H_44_O_11_	*C. oblongifolius*	[112]
**362**	Oblongionoside D	C_24_H_44_O_11_	*C. oblongifolius*	[112]
**363**	Oblongionoside E	C_19_H_36_O_8_	*C. oblongifolius*	[112]
**364**	Oblongionoside F	C_19_H_36_O_8_	*C. oblongifolius*	[112]
**365**	Blumenol A glucoside	C_19_H_30_O_8_	*C. pedicellatus*	[10]
**366**	Sparsioside	C_53_H_102_O_10_	*C. sparsiorus*	[113]
**367**	3,12-dioxo-15,16-epoxy-4*α*-hydroxy-6-(*β*-glucopyranosyl)-ent-neo-clerodan-13(16),14-diene	C_26_H_38_O_10_	*C. limae*	[35]
**368**	Isocrotofolane glucoside	C_26_H_38_O_9_	*C. cascarilloides*	[69]
**369**	2-methoxyphenol-*β*-d-(6-*O*-*β*-d-apiofuranosyl) glucopyranoside	C_18_H_26_O_11_	*C. cascarilloides*	[69]

**Table 21:** Alkaloids from the genus *Croton*.

No.	Compound Name	Molecular Formula	Sources	Ref
**370**	Crotamide A	C_36_H_65_NO	*C. sparsiflorus*	[114]
**371**	Crotamide B	C_38_H_69_NO	*C. sparsiflorus*	[114]
**372**	Crotonine	C_12_H_14_N_2_O_4_	*C. tiglium*	[97]
**373**	Crotonimide A	C_16_H_20_N_2_O_3_	*C. pullei*	[115]
**374**	Crotsparsidine	C_17_H_17_O_3_N	*C. sparsiflorus*	[96]
**375**	Crotonimide C.	C_20_H_20_N_2_O_3_	*C. alienus*	[42]
**376**	6-Hydroxy-1-methyl-2-dimethyl-3,4-tetrahydro-b-carbo-line	C_14_H_19_N_2_O	*C. heliotropiifolius*	[116]
**377**	*N*-*trans*-feruloyl-3,5-dihydroxyindolin-2-one	C_20_H_20_N_2_O_6_	*C. echioides*	[117]

**Table 22:** Benzoate derivatives from the genus *Croton*.

No.	Compound Name	Molecular Formula	Sources	Ref
**378**	2′-(3′’,4′’-dihydroxyphenyl)-ethyl-4-hydroxybenzoate	C_15_H_14_O_5_	*C.* *sylvaticus*	[118]
**379**	3-(4-hydroxy-3,5-dimethoxyphenyl)-propyl benzoate	C_18_H_20_O_5_	*C.* *hutchinsonianus*	[119]
**380**	3-(4-hydroxyphenyl)-propyl benzoate	C_16_H_16_O_3_	*C.* *hutchinsonianus*	[119]

**Table 23:** Pyran-2-one derivatives from the genus *Croton*.

No.	Compound Name	Molecular Formula	Sources	Ref
**381**	Crotonpyrone A	C_17_H_28_O_3_	*C.* *crassifolius*	[120]
**382**	Crotonpyrone B	C_17_H_26_O_3_	*C.* *crassifolius*	[120]
**383**	Crotonpyrone C	C_19_H_28_O_3_	*C.* *crassifolius*	[121]

**Table 24:** Cyclicpeptides from the genus *Croton*.

No.	Compound Name	Molecular Formula	Sources	Ref
**384**	Crotogossamide	C_37_H_56_N_10_O_11_	*C.* *gossypifolius*	[122]
**385**	[1−9-N*α*C]-crourorb A1	C_37_H_56_N_10_O_11_	*C.* *urucurana*	[123]

**Table 25:** Tropone derivatives from the genus *Croton*.

No.	Compound Name	Molecular Formula	Sources	Ref
**386**	Crototropone	C_10_H_12_O_4_	*C.* *zehntneri*	[124]
**387**	Pernambucone	C_15_H_18_O_2_	*C.* *argyroglossum*	[125]

**Table 26:** Limonoids from the genus *Croton*.

No.	Compound Name	Molecular Formula	Sources	Ref
**388**	Musidunin	C_31_H_38_O_11_	*C.* *jatrophoides*	[126]
**389**	Musiduol	C_30_H_38_O_10_	*C.* *jatrophoides*	[126]

**Table 27:** Miscellaneous compounds from the genus *Croton*.

No.	Compound Name	Molecular Formula	Sources	Ref
**390**	Crotoncaudatin	C_22_H_22_O_9_	*C.* *caudatus*	[127]
**391**	8*S*-(−)-8-(4-hydroxy-3-methoxybenzoyl)-dihydrofuran-8(8′*H*)-one	C_20_H_30_O_2_	*C.* *kongensis*	[67]
**392**	Lobaceride	C_35_H_58_O_6_	*C.* *lobatus*	[129]
**393**	Laevifolin A	C_29_H_38_O_4_	*C.* *laevifolius*	[128]
**394**	Laevifolin B	C_29_H_38_O_4_	*C.* *laevifolius*	[128]
**395**	2,6-Dimethyl-1-oxo-4-indanecarboxylic acid	C_12_H_12_O_3_	*C. steenkampianus*	[102]
**396**	3(3′-Methoxy-5′-phenylfuran-2′-yl)propan-1-ol	C_14_H_16_O_3_	*C. oblongifolius*	[22]
**397**	Sparsifol	C_7_H_15_O_6_	*C. sparsiflorus*	[96]
**398**	Sparsioamide	C_43_H_81_NO_5_	*C. sparsiflorus*	[113]
**399**	hexyl Z-ferulate	C_16_H_22_O_4_	*C. laevigatus*	[89]

**Table 28:** Cytotoxic activity of compounds from the genus *Croton*.

Compounds	Tumor Cell Line	Activity (IC_50_)	Ref
Methyl 15,16-epoxy-3,13(16),14-ent-clerodatrien-18,19-olide-17-carboxylate (**6**)	HuCCA-1	36.0 μg/mL	[29]
	KB	26.0 μg/mL	[29]
	HeLa	30.0 μg/mL	[29]
	MDA-MB231	29.0 μg/mL	[29]
	T47D	10.0 μg/mL	[29]
Dimethyl-15,16-epoxy-12-oxo-3,13 (16)14-ent-clerodatriene-17,18-dicarboxylate (**7**)	HuCCA-1	39.0 μg/mL	[29]
	KB	27.0 μg/mL	[29]
	HeLa	29.0 μg/mL	[29]
	MDA-MB231	27.0 μg/mL	[29]
	T47D	25.0 μg/mL	[29]
Laevigatbenzoate (**8**)	HeLa	45.4 μM	[13]
Crotonolide A (**23**)	HL-60	9.42 μM	[21]
	P-388	7.45 μM	[21]
15-oxo-17(10′-*α*-pinenyl)-kauran-18-oic acid (**181**)	HCT-116	7.14 μg/mL	[35]
	OVCAR-8	8.19 μg/mL	[35]
	SF-295	>10.0 μg/mL	[35]
Launine K (**67**)	HeLa	14.5 μM	[37]
	MCF-7	62.5 μM	[37]
Crassin H (**75**)	HL-60	11.8 ± 2.1 μM	[17]
	A549	5.2 ± 0.4 μM	[17]
Crassifolius A (**76**)	Hep3B	17.91 μM	[38]
	HepG2	42.04 μM	[38]
Cracroson D (**339**)	T24	14.48 ± 0.65 μM	[40]
	A549	25.64 ± 2.14 μM	[40]
Cracroson E (**87**)	T24	22.99 ± 1.76 μM	[40]
	A549	51.88 ± 14.07μM	[40]
	Hela	3.9 μM	[48]
	DU145	7.2 μM	[48]
	A549	5.8 μM	[48]
	SGC-7091	13 μM	[48]
	H1975	10 μM	[48]
	HL60	12 μM	[48]
	293T	291.6 μM	[48]
	LX-2	>500.0 μM	[48]
12-*O*-benzoylphorbol-13-(2-methyl)butyrate (**114**)	K562	15 μM	[48]
	MOLT-4	12 μM	[48]
	U937	17 μM	[48]
	MCF-7	20 μM	[48]
	Hela	4.6 μM	[48]
	DU145	4.3 μM	[48]
	A549	6.9 μM	[48]
	SGC-7091	10 μM	[48]
	H1975	3.3 μM	[48]
	HL60	6.8 μM	[48]
	293T	420.4 μM	[48]
	LX-2	>500.0 μM	[48]
12-*O*-tiglyl-7-oxo-5-ene-phorbol-13-(2-methyl)butyrate (**115**)	K562	17 μM	[48]
	MOLT-4	4.8 μM	[48]
	U937	21 μM	[48]
	MCF-7	20 μM	[48]
	Hela	5.0 μM	[48]
	DU145	10 μM	[48]
	A549	19 μM	[48]
	SGC-7091	23 μM	[48]
	H1975	10 μM	[48]
	HL60	10 μM	[48]
	293T	455.3 μM	[48]
	LX-2	>500.0 μM	[48]
13-*O*-(2-metyl)butyryl-4-deoxy-4a-phorbol (**116**)	K562	8.0 μM	[48]
	MOLT-4	9.9 μM	[48]
	U937	18 μM	[48]
	MCF-7	24 μM	[48]
	H1975	10 μM	[48]
	HL60	10 μM	[48]
	293T	455.3 μM	[48]
	LX-2	>500.0 μM	[48]
	Hela	10 μM	[48]
	DU145	10 μM	[48]
	A549	4.5 μM	[48]
	SGC-7091	5.4 μM	[48]
	H1975	3.3 μM	[48]
	HL60	9.8 μM	[48]
	293T	191.0 μM	[48]
	LX-2	>500.0 μM	[48]
Crotignoid A (**117**)	HL-60	1.61 μM	[49]
	A549	2.85 μM	[49]
Crotignoid B (**118**)	HL-60	22.1 μM	[49]
	A549	31.0 μM	[49]
Crotignoid C (**119**)	HL-60	32.3 μM	[49]
	A549	5.03 μM	[49]
Crotignoid D (**120**)	HL-60	19.8 μM	[49]
	A549	10.2 μM	[49]
Crotignoid F (**122**)	HL-60	44.6 μM	[49]
	A549	6.96 μM	[49]
Crotignoid G (**123**)	HL-60	22.1 μM	[49]
	A549	3.89 μM	[49]
Crotignoid H (**124**)	HL-60	9.97 μM	[49]
	A549	8.08 μM	[49]
Crotignoid I (**125**)	HL-60	14.8 μM	[49]
	A549	24.4 μM	[49]
Crotignoid J (**126**)	HL-60	14.2 μM	[49]
	A549	29.5 μM	[49]
Crotusin A (**128**)	HL-60	12.53 ± 0.37 μM	[44]
	SMMC-7721	7.06 ± 0.72 μM	[44]
	A549	9.69 ± 0.41 μM	[44]
	MCF-7	9.56 ± 0.76 μM	[44]
	SW480	14.88 ± 0.43 μM	[44]
Crotusin B (**129**)	HL-60	19.39 ± 0.46 μM	[44]
	SMMC-7721	21.13 ± 0.29 μM	[44]
	A549	14.66 ± 1.66 μM	[44]
	MCF-7	1.49 ± 0.23 μM	[44]
	SW480	31.21 ± 3.20 μM	[44]
Crotusin C (**130**)	HL-60	4.19 ± 0.15 μM	[44]
	SMMC-7721	3.87 ± 0.12 μM	[44]
	A549	2.44 ± 0.35 μM	[44]
	MCF-7	0.49 ± 0.04 μM	[44]
	SW480	2.89 ± 0.01 μM	[44]
12-*O*-tiglylphorbol-4-deoxy-4*β*-phorbol-13-acetate (**131**)	SNU387	59.5 ± 2.1 μM	[50]
	SNU398	43.7 ± 1.5 μM	[50]
12-*O*-tiglylphorbol-4-deoxy-4*β*-phorbol-13-hexadecanoate (**132**)	SNU387	30.2 ± 1.4 μM	[50]
	SNU398	91.2 ± 3.7 μM	[50]
13-*O*-acetylphorbol-4-deoxy-4*β*-phorbol-20-oleate (**133**)	SNU387	1.9 ± 0.2 μM	[50]
	SNU398	13.5 ± 1.1 μM	[50]
13-*O*-acetylphorbol-4-deoxy-4*β*-phorbol-20-linoleate (**134**)	SNU387	0.71 ± 0.08 μM	[50]
	SNU398	18.2 ± 1.7 μM	[50]
4-deoxy-20-oxophorbol 12-tiglyl 13-acetate (**135**)	K562	0.03 μM	[51]
	A549	6.88 μM	[51]
	Huh-7	3.85 μM	[51]
7-oxo-5-ene-phorbol-13-(2-methylbutyrate) (**136**)	K562	0.03 μM	[51]
	A549	6.33 μM	[51]
	Huh-7	20.9 μM	[51]
7-hydroxyl-phorbol-5-ene-13-(2-methyl)butyrate (**137**)	K562	0.07 μM	[51]
	A549	8.86 μM	[51]
	Huh-7	11.6 μM	[51]
13-*O*-(2-metyl)butyryl-phorbol (**139**)	K562	0.05 μM	[51]
	A549	43.5 μM	[51]
	Huh-7	34.2 μM	[51]
7-keto-12-*O*-tiglylphorbol-13-acetate (**140**)	HL-60	6.22 ± 3.24 μg/mL	[52]
	A549	18.0 ± 9.48 μg/mL	[52]
Phorbol-13-isobutyrate (**148**)	HL-60	0.22 ± 0.15 μg/mL	[52]
14-*epi*-hyalic acid (**159**)	HL-60	8.2 μM	[63]
Kongeniod A (**178**)	HL-60	1.27 ± 0.24 μM	[59]]
	A549	5.74 ± 0.25 μM	[59]
Kongeniod B (**179**)	HL-60	0.47 ± 0.04 μM	[59]
	A549	3.25 ± 0.91 μM	[59]
Kongeniod C (**180**)	HL-60	0.58 ± 0.17 μM	[59]
Crotonkinensin D (**188**)	MCF-7	9.4 ± 1.7 μM	[61]
	MCF-7/TAMR	2.6 ± 0.9 μM	[61]
	MCF-7/ADR	18.9 ± 0.6 μM	[61]
	MDA-MB-231	22.0 ± 0.9 μM	[61]
EBC-162 (**207**)	HL-60	15 μg/mL	[74]
	HT29	15 μg/mL	[74]
	MCF-7	30 μg/mL	[74]
	MM96	10 μg/mL	[74]
	NNF	20 μg/mL	[74]
	K562	50 μg/mL	[74]
EBC-233 (**208**)	HL-60	10 μg/mL	[74]
	HT29	80 μg/mL	[74]
	MCF-7	20 μg/mL	[74]
	MM96	6 μg/mL	[74]
	NNF	50 μg/mL	[74]
	K562	50 μg/mL	[74]
EBC-300 (**209**)	HL-60	35 μg/mL	[74]
	HT29	100 μg/mL	[74]
	MCF-7	100 μg/mL	[74]
	MM96	80 μg/mL	[74]
	NNF	80 μg/mL	[74]
	K562	100 μg/mL	[74]
EBC-240 (**210**)	HL-60	45 μg/mL	[74]
	HT29	80 μg/mL	[74]
	MCF-7	50 μg/mL	[74]
	MM96	12 μg/mL	[74]
	NNF	80 μg/mL	[74]
	K562	60 μg/mL	[74]
EBC-241 (**211**)	HL-60	40 μg/mL	[74]
	HT29	80 μg/mL	[74]
	MCF-7	40 μg/mL	[74]
	MM96	12 μg/mL	[74]
	NNF	75 μg/mL	[74]
	K562	60 μg/mL	[74]
Furanocembranoid 1 (**266**)	BT474	7.8 μg/mL	[83]
	CHAGO	7.0 μg/mL	[83]
	Hep-G2	5.6 μg/mL	[83]
	KATO-3	5.9 μg/mL	[83]
	SW-620	6.3 μg/mL	[83]
Furanocembranoid 2 (**267**)	BT474	9.5 μg/mL	[83]
	CHAGO	>10 μg/mL	[83]
	Hep-G2	>10 μg/mL	[83]
	KATO-3	6.8 μg/mL	[83]
	SW-620	9.9 μg/mL	[83]
Furanocembranoid 3 (**268**)	BT474	9.6 μg/mL	[83]
	CHAGO	7.1 μg/mL	[83]
	Hep-G2	5.7 μg/mL	[83]
	KATO-3	8.2 μg/mL	[83]
	SW-620	5.6 μg/mL	[83]]
Furanocembranoid 4 (**269**)	BT474	9.6 μg/mL	[83]
	CHAGO	9.3 μg/mL	[83]
	Hep-G2	6.1 μg/mL	[83]
	KATO-3	8.1 μg/mL	[83]
	SW-620	6.0 μg/mL	[83]
Laevigatlactone B (**272**)	Hela	38.4 μM	[84]
(+)-[1R*,2S*,7S*,8S*,12R*]-7,8-Epoxy-2,12-cyclocembra-3*E*,10*Z*dien-20,10-olide (**276**)	PEO1	132 nM	[85]
	PEO1TaxR	200 nM	[85]
(+)-[1R*,4S*,10R*]-4-Hydroxycembra-2*E*,7*E*,11*Z*-trien-20,10-olide (**278**)	PEO1	125 nM	[85]
	PEO1TaxR	135 nM	[85]
Crotontomentosin A (**294**)	Hela	24.0 ± 2.6 μM	[88]
	Hep G2	87.9 ± 4.5 μM	[88]
	MDA-MB-231	54.1 ± 2.1 μM	[88]
	A549	40.6 ± 3.9 μM	[88]
Crotontomentosin B (**295**)	Hela	>100 μM	[88]
	Hep G2	28.1 ± 2.1 μM	[88]
	MDA-MB-231	28.7 ± 3.4 μM	[88]
	A549	29.1 ± 5.2 μM	[88]
Crotontomentosin C (**297**)	Hela	47.9 ± 3.3 μM	[88]
	Hep G2	83.3 ± 5.3 μM	[88]
	MDA-MB-231	>100 μM	[88]
	A549	>100 μM	[88]]
Crotontomentosin D (**296**)	Hela	59.7 ± 4.5 μM	[88]
	Hep G2	>100 μM	[88]
	MDA-MB-231	49.3 ± 2.8 μM	[88]
	A549	>100 μM	[88]
Crotolaevigatone B (**300**)	A549	21.2 μM	[89]
	MDA-MB-231	33.4 μM	[89]
Crotolaevigatone G (**305**)	A549	25.6 μM	[89]
	MDA-MB-231	32.7 μM	[89]
EBC-324 (**311**)	MCF-7	40 μM	[92]
	NFF	50 μM	[92]
	K562	6 μM	[92]
EBC-329 (**312**)	MCF-7	13 μM	[92]
	NFF	40 μM	[92]
	K562	0.6 μM	[92]
*ent*-3*β*-hydroxypimara-8(14),9,15-trien-12-one (**319**)	NFF	23 μg/mL	[98]
	Hela	13 μg/mL	[98]
	HT 29	13 μg/mL	[98]
	MCF-7	16 μg/mL	[98]
	MM96L	2.8 μg/mL	[98]
	K562	17 μg/mL	[98]
EBC-325 (**321**)	MCF-7	20 μM	[99]
	NFF	6 μM	[99]
	K562	3 μM	[99]
EBC-326 (**322**)	MCF-7	14 μM	[99]
	NFF	6 μM	[99]
	K562	6 μM	[99]
EBC-327 (**323**)	MCF-7	10 μM	[99]
	NFF	10 μM	[99]
	K562	10 μM	[99]
3-hydroxycleistantha-13(17),15-diene (**325**)	KATO-3	6.0 μg/mL	[93]
	SW-620	>10 μg/mL	[93]
	BT474	6.1 μg/mL	[93]
	Hep-G2	0.5 μg/mL	[93]
	CHAGO	5.5 μg/mL	[93]
3,4-*seco*-cleistantha-4(18),13(17),15-trien-3-oic acid (**326**)	KATO-3	9.6 μg/mL	[93]
	SW-620	>10 μg/mL	[93]
	BT474	10 μg/mL	[93]
	Hep-G2	8.6 μg/mL	[93]
	CHAGO	>10 μg/mL	[93]
Crotobarin (**330**)	KB	2.5 ± 0.10 μM	[101]
	HT29	2.1 ± 0.60 μM	[101]
	A549	0.79 ± 0.15 μM	[101]
	HL60	0.56 ± 0.02 μM	[101]
Crotogoudin (**331**)	KB	1.5 ± 0.03 μM	[101]
	HT29	1.9 ± 0.25 μM	[101]
	A549	0.54 ± 0.02 μM	[101]
	HL60	0.49 ± 0.01 μM	[101]
Crotonpyrone A (**381**)	Hela	10.21 μg/mL	[120]
	NCI-446	6.59 μg/mL	[120]
Crotonpyrone B (**382**)	Hela	9.54 μg/mL	[120]
[1−9-N*α*C]-crourorb A1 (**385**)	NCI-ADR/RES	4.8 μM	[123]
